# Relative Gains, Losses, and Reference Points in Probabilistic Choice in Rats

**DOI:** 10.1371/journal.pone.0117697

**Published:** 2015-02-06

**Authors:** Andrew T. Marshall, Kimberly Kirkpatrick

**Affiliations:** Department of Psychological Sciences, Kansas State University, Manhattan, Kansas, United States of America; University of Leicester, UNITED KINGDOM

## Abstract

Theoretical reference points have been proposed to differentiate probabilistic gains from probabilistic losses in humans, but such a phenomenon in non-human animals has yet to be thoroughly elucidated. Three experiments evaluated the effect of reward magnitude on probabilistic choice in rats, seeking to determine reference point use by examining the effect of previous outcome magnitude(s) on subsequent choice behavior. Rats were trained to choose between an outcome that always delivered reward (low-uncertainty choice) and one that probabilistically delivered reward (high-uncertainty). The probability of high-uncertainty outcome receipt and the magnitudes of low-uncertainty and high-uncertainty outcomes were manipulated within and between experiments. Both the low- and high-uncertainty outcomes involved variable reward magnitudes, so that either a smaller or larger magnitude was probabilistically delivered, as well as reward omission following high-uncertainty choices. In Experiments 1 and 2, the between groups factor was the magnitude of the high-uncertainty-smaller (H-S) and high-uncertainty-larger (H-L) outcome, respectively. The H-S magnitude manipulation differentiated the groups, while the H-L magnitude manipulation did not. Experiment 3 showed that manipulating the probability of differential losses as well as the expected value of the low-uncertainty choice produced systematic effects on choice behavior. The results suggest that the reference point for probabilistic gains and losses was the expected value of the low-uncertainty choice. Current theories of probabilistic choice behavior have difficulty accounting for the present results, so an integrated theoretical framework is proposed. Overall, the present results have implications for understanding individual differences and corresponding underlying mechanisms of probabilistic choice behavior.

## Introduction

The analysis of risk-sensitive decision making has become a well-established area of research in fields such as human judgment and decision making, animal choice behavior, and neuroeconomics [1,2]. Probabilistic choice tasks typically involve choices between two options: a low-uncertainty smaller magnitude outcome and a high-uncertainty larger magnitude outcome [3]. Manipulations of the probability and magnitude of the high-uncertainty outcome have been shown to considerably impact probabilistic choice behavior in humans [3–5]. Previous research has shown that individual differences in probabilistic choice behavior are related to gambling [6], cigarette smoking [7], and percent body fat [8]. These results are especially relevant given the prevalence of risky behaviors such as pathological gambling [9,10] and drug use and abuse [11]. Ultimately, these data reflect the necessity to determine the psychological and neurobiological mechanisms of probabilistic decision making via the improved understanding of adequate animal models of such behaviors [12–15].

One of the inherent properties of probabilistic decision making that has been overlooked in the development of animal models is the presence of and sensitivity to differential gains and losses. Gains and losses are judged relative to subjective reference points [16–19], in which positive and negative deviations from the reference point equate to subjective gains and losses, respectively. One possible reference point is the individual’s current state, or what an individual currently has [16]. This conceptualization is reasonable in regards to the human probabilistic choice literature, as the monetary rewards that have been traditionally used as probabilistic choice outcomes can be easily decreased or increased. However, given the use of consumable rewards within the animal choice literature, the treatment of gains and losses relative to a current state is challenging to operationalize, as an experimenter cannot readily take food away because it will have already been consumed in most cases. Accordingly, the criterion distinguishing gains from losses in non-human animals has been proposed to take on other values, such as the expected value of a choice [20]. However, despite previous research that has identified both differential effects of gains and losses on probabilistic choice behavior in non-human animals [21] and important roles for gains and losses (relative to a reference point) in human decision making [16,22,23], explicit reference point use in rats remains open for investigation. Accordingly, the goal of the present study was to identify reference point use in rats, an important pre-clinical model for understanding human risky decision making [15].

Indeed, the framing of outcomes as gains or losses considerably influences behavior in both human and non-human animals [16,24–26]. The corresponding effects of differential gains and losses may be elucidated via trial-by-trial analysis of choice behavior, as the outcomes of previous choices have been shown to affect subsequent decision making behaviors in several species [27–35]. One example of these local influences on choice behavior is a tendency to repeat the same behavior following a gain (i.e., win-stay), coupled with a reduced propensity to repeat the same behavior following a loss (i.e., lose-shift) [21,28,31,33,36–40]. Here, lose-shift behavior is operationally defined as a *relatively* decreased rate of repeating the same behavior, as previous research has also demonstrated relatively more shifting (i.e., less staying) behavior following losses relative to gains [21]; indeed, subjective biases may interfere with staying versus shifting behavior or a tendency to explore versus exploit various outcomes, such that animals may stay with the same choice after experienced losses. Interestingly, many risky-choice situations, including casino games [41], do not adjust the probability of winning (or losing) as a function of the most recent outcome, so a rational decision maker should not be differentially affected by whether the previous outcome was a gain or a loss [42]. Nevertheless, the strong effect of the previous gains and losses suggests that human and non-human decision makers are considerably influenced by such factors within probabilistic choice tasks, and that the corresponding psychological processes warrant further investigation.

While the effects of previous outcomes on subsequent probabilistic choice have been frequently investigated [21], the mechanisms by which animals track the outcomes as gains and losses have not been conclusively determined. For example, if given the choice between an outcome that always delivers one pellet (i.e., low-uncertainty) and an outcome of high-uncertainty that probabilistically delivers zero or four pellets [21], the high-uncertainty four-pellet outcome may be considered as a gain relative to several possible reference points. Rats could potentially use the high-uncertainty expected value, the zero-outcome on the high-uncertainty side, and/or the low-uncertainty expected value as reference points.

If rats used a *high-uncertainty reference point* to gauge specific high-uncertainty magnitudes as gains or losses, then the four-pellet high-uncertainty magnitude in the preceding example would be judged as a gain because it is greater than the expected (mean) value of the high-uncertainty outcome, and the zero-pellet magnitude would be judged as a loss because it is less than the expected value of the high-uncertainty outcome. Note that the expected value of a choice would presumably be learned through experience with choices and outcomes. In this example, rats should show win-stay behavior follow the four-pellet outcome and lose-shift behavior following the zero-pellet outcome. Previous theoretical approaches to probabilistic choice behavior, such as prospect theory [16] and reinforcement learning [20], have posited the expected value of a choice as a potential reference point for determining the gain-loss value of outcomes for that choice, consistent with the possibility that rats may use a high-uncertainty reference point to guide subsequent high-uncertainty choices.

An alternative possible reference point is a *zero-outcome reference point*. In this case, all non-zero magnitudes would be regarded as gains due to compensation of energy expended while responding for that choice. The omission of food reward associated with the zero-pellet outcome would be a net loss due to wasted energy expenditure. Optimal foraging theory has proposed a gain-loss criterion similar to a zero-outcome reference point, in which the encoding of gains and losses reflects the minimum daily energy intake required for survival [43]; the failure to approach this minimum value (i.e., receiving zero reward for a choice) would be considered as a loss. Here, a loss (or gain) represents an outcome that is less than (or greater than) the current status quo. Thus, if a zero-pellet outcome is the subjective reference point, then the rats should exhibit win-stay behavior following all non-zero food amounts and lose-shift behavior following the zero-pellet outcome.

A third potential reference point is a *low-uncertainty reference point*. In this case, high-uncertainty gains and losses may be regarded relative to the alternative low-uncertainty choice. While this specific idea for a reference point has, to our knowledge, not been previously considered in the encoding of gains and losses in probabilistic choice in non-human animals, it has received some attention in the human literature [44,45], in examining the subjective regret produced when an uncertain outcome yields a poorer result than what could have been achieved through a different alternative. Interestingly, recent research has suggested that rats may experience regret. Specifically, Steiner and Redish [46] showed that when foregone low-cost outcomes are immediately followed by a possible high-cost outcome, rats show behavioral and neurobiological activity that is potentially consistent with the experience of regret [47]. While such results may reflect outcome valuation relative to an alternative outcome (i.e., low-uncertainty reference point), the task involved costs that were determined by expected delays until reward as opposed to the experienced probabilistic outcome magnitudes. Furthermore, the results seemingly identified that rats *could* experience regret, rather than explicitly identifying that such regret underlies the subjective criterion categorizing gains and losses (i.e., in terms of an alternative-outcome reference point). Therefore, it is critical to evaluate choice behavior within probabilistic decision making tasks in which outcome magnitude may be inherently dynamic given the task employed. If high-uncertainty gains and losses are compared to a low-uncertainty reference point, then rats should exhibit win-stay and lose-shift behavior, respectively, following high-uncertainty food rewards greater than and less than the expected value of the low-uncertainty choice.

Given that a food outcome may be regarded as a gain or loss relative to *high-uncertainty*, *zero-outcome*, or *low-uncertainty* reference points, it is important to design experiments to differentiate between these possibilities. Accordingly, the goal of the present set of experiments was to determine the reference points that distinguish gains from losses in rats by analyzing the effects of low-uncertainty and high-uncertainty choice outcome magnitudes on global and local probabilistic choice behavior. The primary criterion for reference-point identification (and thus the identification of gains and losses) was the presence of considerable differences in choice behavior following differential outcomes [19]. The current paradigm was similar to that used by Marshall and Kirkpatrick [33], in which both low-uncertainty choices and rewarded high-uncertainty choices involved variable-magnitude outcomes. The use of variable outcomes, in conjunction with probabilistic food omissions following high-uncertainty choices, permitted differentiation between different reference points and a greater understanding of the mechanisms governing choice behavior following experienced gains and losses. Experiments 1 and 2 investigated the effect of individual probabilistic outcome magnitudes on global and local choice behavior to determine which outcomes were gauged as probabilistic gains and/or losses. Subsequently, Experiment 3 determined the effect of manipulations of differential probabilistic losses on choice behavior, in addition to evaluating the role of the low-uncertainty outcome choice in its effect on probabilistic choice.

## Experiment 1

### Method

**Animals**. Twenty-four experimentally-naive male Sprague-Dawley rats (Charles River, Portage, MI) were used in the experiment. They arrived at the facility (Kansas State University, Manhattan, KS) at approximately 45 days of age. The rats were pair-housed in a red-illuminated colony room that was set to a reverse 12:12 hr light:dark schedule (lights off at approximately 6 am). The rats were tested during the dark phase. There was ad libitum access to water in the home cages and in the experimental chambers. The rats were maintained at approximately 85% of their projected ad libitum weight during the experiment, based on growth-curve charts obtained from the supplier. When supplementary feeding was required following an experimental session, the rats were fed in their home cages approximately 1 hr after being returned to the colony room [48,49].

**Ethics statement**. All research was conducted in accordance with the Guide for the Care and Use of Laboratory Animals of the National Research Council, and with the approval of the Kansas State University Institutional Animal Care and Use Committee (Protocol 3102).

**Apparatus**. The experiment was conducted in 24 operant chambers (Med-Associates, St. Albans, VT) each housed within sound-attenuating, ventilated boxes (74 × 38 × 60 cm). Each operant chamber (25 × 30 × 30 cm) was equipped with a stainless steel grid floor; two stainless steel walls (front and back); and a transparent polycarbonate side wall, ceiling, and door. Two pellet dispensers (ENV-203), mounted on the outside of the operant chamber, delivered 45-mg food pellets (Bio-Serv, Frenchtown, NJ) to a food cup (ENV-200R7) that was centered on the lower section of the front wall. Head entries into the food magazine were transduced by an infrared photobeam (ENV-254). Two retractable levers (ENV-112CM) were located on opposite sides of the food cup. Water was always available from a sipper tube that protruded through the back wall of the chamber. Experimental events were controlled and recorded with 2-ms resolution by the software program MED-PC IV [50].

### Procedure

**Magazine and lever press training**. The rats were given a random-time 60-s schedule of food deliveries for magazine training, earning approximately 120 pellets in a single 2-hr session. The rats were then given two sessions of lever-press training with a fixed ratio (FR) 1 schedule of reinforcement that was followed by a random ratio (RR) 3 schedule and then an RR 5; each of these schedules lasted until the rats earned 20 pellets on each lever.

**Probabilistic choice task**. Each pair of rats was randomly assigned to one of three groups (*n* = 8). Following a rewarded high-uncertainty choice, Group 1–11 received 1 or 11 pellets, Group 2–11, 2 or 11 pellets, and Group 4–11, 4 or 11 pellets (p = .50). The smaller and larger high-uncertainty choices were labeled the high-small (H-S) and high-large (H-L) outcomes, respectively. An unrewarded high-uncertainty choice resulted in no food being delivered (high-zero, H-Z). The alternative low-uncertainty choice resulted in either 2 or 4 pellets (p = .50; low-small, L-S, and low-large, L-L, respectively).

Each session consisted of 8 forced choice trials followed by a series of free choice trials. On forced choice trials, one lever was inserted into the chamber. Each lever corresponded to one of two choices; lever assignments were counterbalanced within each pair of rats. When the lever was pressed, a fixed interval 20-s schedule began; the first lever press after 20 s resulted in lever retraction and food delivery. If the lever corresponded to the low-uncertainty outcome, then either the L-S or L-L outcome was delivered (p = .50). If the forced choice lever corresponded to the high-uncertainty outcome, then either the H-S or H-L outcome was delivered (p = .50). In forced choice trials, food was always delivered following high-uncertainty forced choices. Each non-zero food amount for the low-uncertainty and high-uncertainty choices was presented twice in the forced choice trials in a random order. Free choice trials were identical to forced choice trials except that two levers were inserted into the chamber, a choice on one of the levers caused the other lever to retract, and food omission was possible following high-uncertainty choices (i.e., H-Z). A 10-s inter-trial interval (ITI) intervened between successive trials.

In different phases, the programmed probability of food delivery following a high-uncertainty choice was .10, .25, .33, .50, .67, .75, or .90 (see Table 1 for high-uncertainty choice expected values for each group). Food deliveries on individual trials were determined using a probability with replacement algorithm so that individual trial outcomes were orthogonal to each other. All rats were first exposed to high-uncertainty food deliveries with a probability of .50. Phase 2 involved a reversal of the lever assignments to reduce any lever biases. For Phases 3–8, the rats in each group were partitioned into two subgroups (based on their high-uncertainty choice behavior in Phase 2) that experienced the probabilities of high-uncertainty food delivery in different orders: .75, .33, .67, .25, .90, and .10; or .25, .67, .33, .75, .10, and .90. In Phase 9, all of the rats were returned to the probability of .50.

**Table 1:** Expected value of the high-uncertainty choice as a function of the probability of food for a high-uncertainty choice for each group in Experiment 1.

Probability	Group 1–11	Group 2–11	Group 4–11
.10	0.60	0.65	0.75
.25	1.50	1.63	1.88
.33	2.00	2.17	2.50
.50	3.00	3.25	3.75
.67	4.00	4.33	5.00
.75	4.50	4.88	5.63
.90	5.40	5.85	6.75

Group 1–11 received 1 or 11 pellets following a high-uncertainty choice, Group 2–11, 2 or 11 pellets, and Group 4–11, 4 or 11 pellets.

Phases 1 and 2 lasted for 20 sessions each. Phase 3 lasted for 22 sessions. After the first 12 sessions of Phase 3, the maximum number of free choice trials was reduced from 160 to 100 to better maintain the rats’ weights at approximately 85% of their ad libitum weights; this change was maintained for the rest of the experiment. Phase 4 lasted for 11 sessions. Phases 5–9 lasted for 10 sessions each. Each session terminated after all free choice trials were completed or after 2 hr.

**Data analysis**. The final five sessions of Phases 3–9 were used for data analyses, with the exception of one rat that did not complete Phase 9; for this rat, the first 100 choice trials of each of the final five sessions in Phase 2 were used. The rats’ molar choice behavior was stable across the final five sessions of each phase. Across Phases 2–9, the mean absolute deviation in choice percentages between each of the final five sessions and the mean of the final five sessions of each phase was 4.5%. Statistical analyses were collapsed across the different orders of exposure to the probabilities of uncertain food delivery. Specifically, an ANOVA with factors of group (1–11, 2–11, 4–11) and order of probability exposure was conducted on choice data from Phase 2 and Phase 9 (i.e., when the probability of high-uncertainty food equaled .50). There were no significant Group × Order or Phase × Group × Order interactions, suggesting that the order of probabilities did not interact with any effect of group (i.e., an effect of H-S reward magnitude), the primary effect of interest. Lastly, only significant test statistics are reported, *p* < .05. Unless described otherwise, post-hoc tests with a Bonferroni correction (with original alpha value of .05 prior to correction) were employed for the analyses of significant effects.

Molar analyses of choice behavior involved analyzing the log odds of high-uncertainty choices. The log odds of a choice is a more sensitive measure to detect differences in choice behavior, reduces ceiling and floor effects, and more readily meets the data scaling assumptions of the general linear model employed for analysis of variance (ANOVA). The empirical log odds formulation was of the following form:

$$LogOdds=\log\frac{N_{H}+0.5}{N_{L}+0.5}$$

in which *N*_*H*_ was the number of high-uncertainty choices and *N*_*L*_ was the number of low-uncertainty choices [51,52]. A value of 0.5 was added to the numerator and denominator to correct for exclusive choice behavior [53]. Here, values greater than zero reflect more choices for the option in the numerator (i.e., high-uncertainty choices), while values less than zero reflect more choices for the option in the denominator (i.e., low-uncertainty choices).

Molecular analyses involved measuring the log odds of high-uncertainty choices as a function of the previous outcome. These data were collapsed across the probability of high-uncertainty choice food delivery. As described previously [33], when the number of high-uncertainty choices was small (i.e., at low probabilities of high-uncertainty food), there was the possibility that an animal never received one of the high-uncertainty magnitudes due to the reduction in the number of corresponding choices. Thus, there would be no data as a function of a previous high-uncertainty outcome because that outcome was never delivered. Accordingly, these data were collapsed across probabilities due to such missing data within some probability-outcome combinations; for analyses of previous outcome by probability interactions on choice, see [33].

For both molar and molecular analyses, the internal reliability of individual differences was measured using Cronbach’s alpha (α). This provides an assessment of consistency in choice behavior of individuals across testing with different probabilities of high-uncertainty food.

### Results

**Fig 1:**
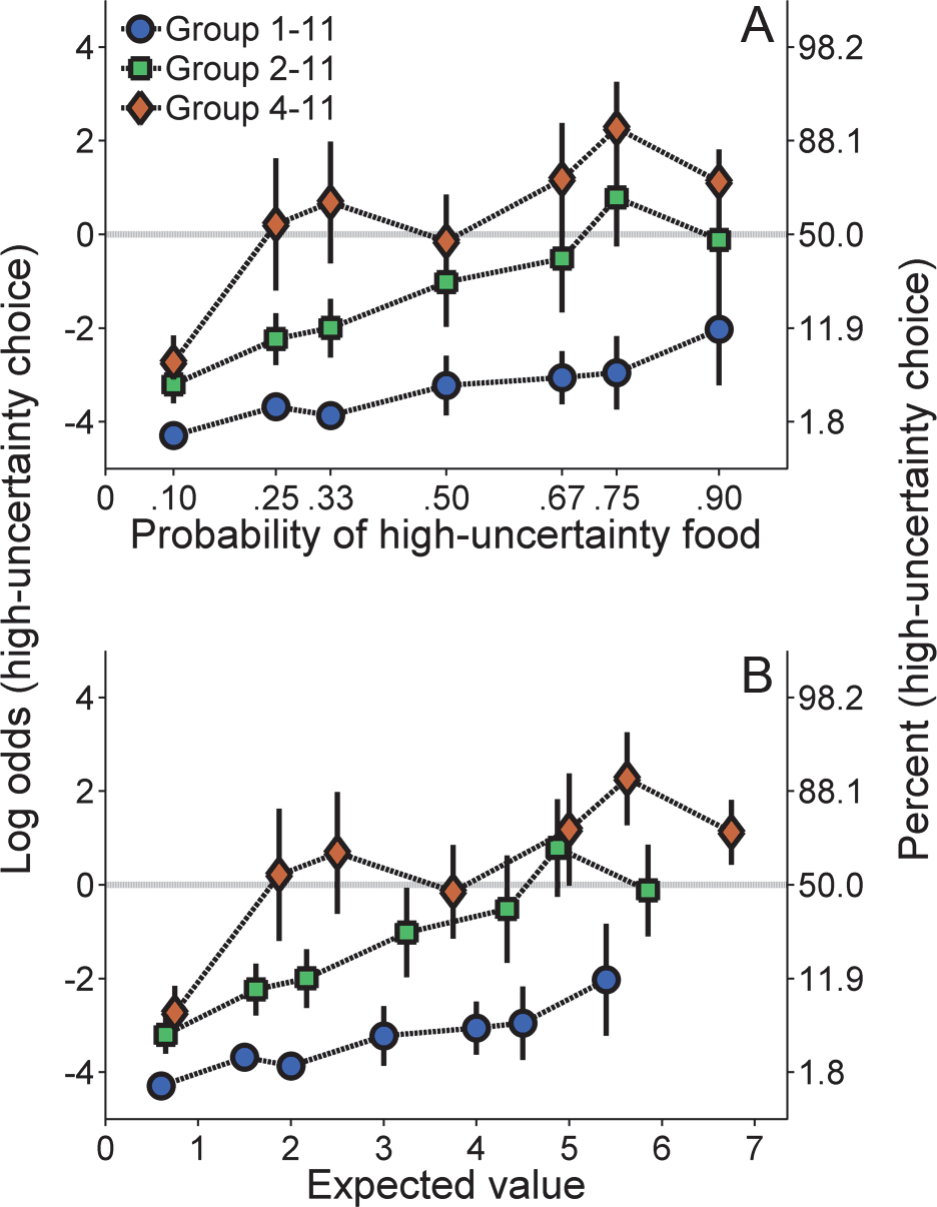
Mean (±SEM) log odds of high-uncertainty choices for each group in Experiment 1 as a function of the probability of high-uncertainty food delivery (A) and the expected value of the high-uncertainty outcome (B). The horizontal line indicates neutral preference of the two outcomes. Values below this line reflect risk aversion (more low-uncertainty than high-uncertainty choices), and values above it reflect risk proneness (more high-uncertainty than low-uncertainty choices). A second ordinate showing the percentages corresponding to the log odds values was included to aid interpretation.

**Molar analysis**. Fig. 1A shows the log odds of high-uncertainty choices as a function of the probability of high-uncertainty choice food delivery. All groups exhibited a general increase in high-uncertainty choices as the probability of food increased. Furthermore, Group 4–11 made the most high-uncertainty choices, followed by Group 2–11 and then Group 1–11.

Choice behavior was stable across probabilities of food, α = .92. An ANOVA with probability as the within groups factor and group (1–11, 2–11, and 4–11) as the between groups factor revealed main effects of probability, *F*(6, 126) = 10.73, *p* < .001, η_p_^2^ = .338, and group, *F*(2, 21) = 7.64, *p* = .003, η_p_^2^ = .421. Post-hoc analyses revealed that Group 1–11 chose the high-uncertainty outcome significantly less than Group 4–11. To determine whether the effect of the H-S reward magnitude on choice behavior was due to the different expected values at each probability of high-uncertainty food delivery, the choice results are plotted as a function of the high-uncertainty expected value in Fig. 1B (see Table 1 for expected values). At similar expected values of the high-uncertainty choice, Group 1–11 still made high-uncertainty choices less often than Group 2–11, which chose the high-uncertainty outcome less than Group 4–11. In addition, Groups 1–11 and 2–11 were overly risk averse. The predicted indifference point for choices (where the log odds would equal 0) should be at an expected value of 3, which is equal to the low-uncertainty choice expected value. Group 4–11 displayed indifference near this expected value and preferred the high-uncertainty choice for higher expected values, but the other two groups remained biased towards the low-uncertainty choice.

**Fig 2:**
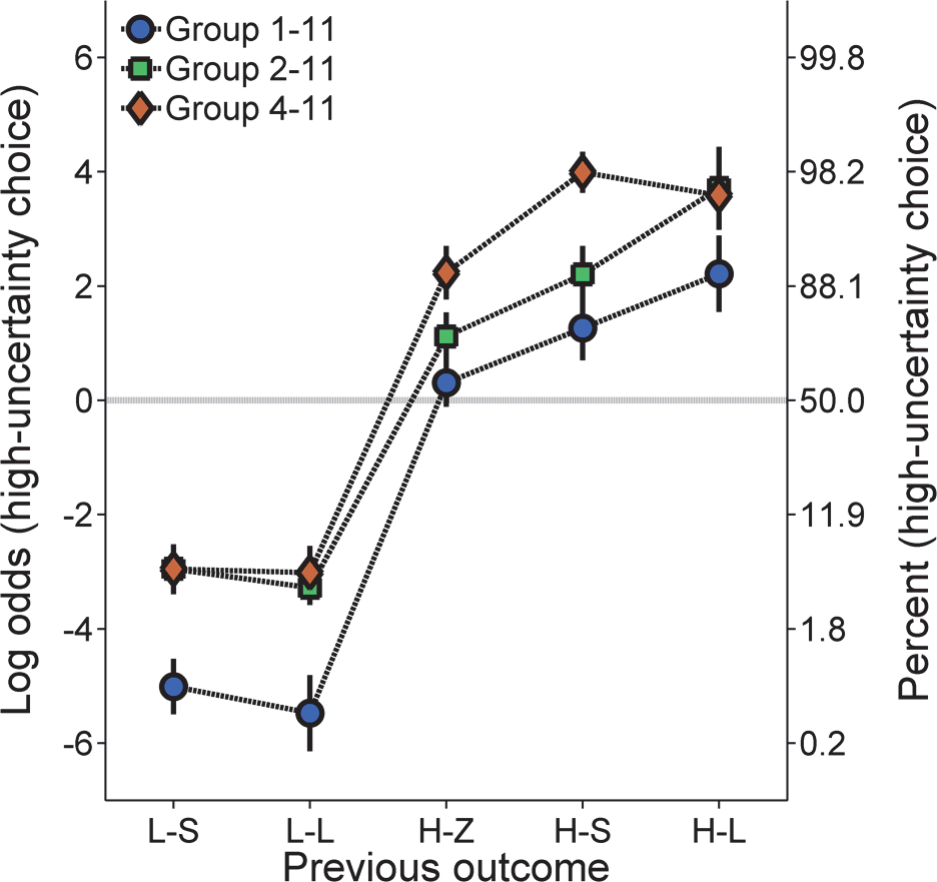
Mean (±SEM) log odds of high-uncertainty choices for each group in Experiment 1 as a function of the outcome of the previous choice, collapsed across the probability of high-uncertainty food delivery. A second ordinate showing percentages corresponding to the log odds values was included to aid interpretation. L-S = low-uncertainty-small; L-L = low-uncertainty-large; H-Z = high-uncertainty-zero; H-S = high-uncertainty-small; H-L = high-uncertainty-large.

**Molecular analysis**. Fig. 2 shows the log odds of high-uncertainty choices as a function of the previous outcome. Following L-S and L-L outcomes, the rats were more likely to stay on the low-uncertainty side than they were to switch to the high-uncertainty side. Following H-Z, H-S, and H-L outcomes, the rats were more likely to make another high-uncertainty choice. Individual differences in molecular choice behavior were stable as a function of the previous outcome, α = .85. An ANOVA with previous outcome as the within groups factor and group as the between groups factor revealed main effects of previous outcome, *F*(4, 84) = 225.55, *p* < .001, η_P_^2^ = .915, and group, *F*(2, 21) = 9.73, *p* = .001, η_P_^2^ = .481. Post-hoc analyses revealed that Group 1–11 chose the high-uncertainty outcome significantly less often than Groups 2–11 and 4–11. Furthermore, high-uncertainty choice behavior following L-S and L-L outcomes was significantly less than following H-Z, H-S, and H-L outcomes and uncertain choice behavior following H-Z outcomes was significantly less than following H-S and H-L outcomes. Choice behavior following L-S versus L-L outcomes or following H-S versus H-L outcomes did not significantly differ.

In conjunction with our hypotheses and specific goals concerning the distinction of gains from losses, additional post-hoc analyses were performed to target possible group differences in choice behavior following H-S and H-L outcomes. Significant differences in subsequent choice behavior following H-S and H-L outcomes may suggest that these outcomes were regarded as losses and gains, respectively, and that the group differences were due to exposure to differential gains and losses. An ANOVA with previous outcome (H-S, H-L) as the within groups factor and group as the between groups factor revealed a significant increase in the log odds of high-uncertainty choices following H-L relative to H-S outcomes, *F*(1, 21) = 8.03, *p* = .010, η_P_^2^ = .277, a main effect of group, *F*(2, 21) = 4.23, *p* = .029, η_P_^2^ = .287, and a Previous Outcome × Group interaction, *F*(2, 21) = 5.52, *p* = .012, η_P_^2^ = .344. Paired sample *t*-tests conducted on the interaction revealed that Groups 1–11 and 2–11 were significantly less likely to make a high-uncertainty choice following H-S outcomes than following H-L outcomes.

**Fig 3:**
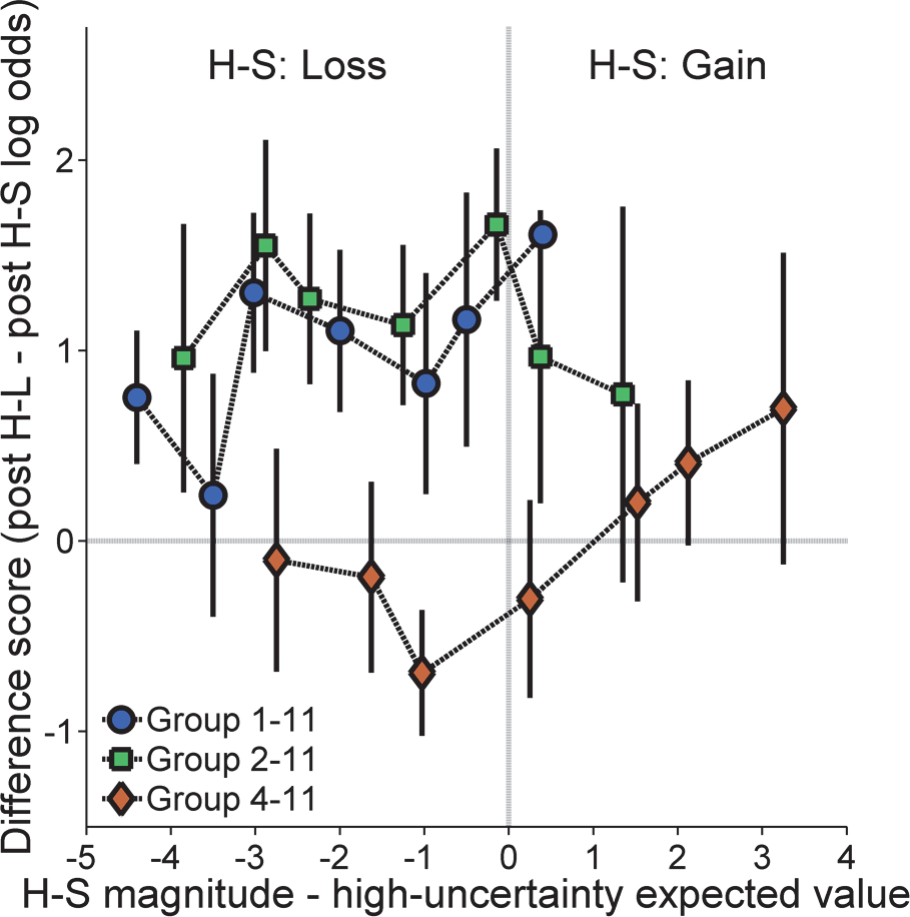
Mean (±SEM) difference score between the log odds of high-uncertainty choices following high-uncertainty-large (H-L) outcomes and the log odds of high-uncertainty choices following high-uncertainty-small (H-S) outcomes for each group in Experiment 1 as a function of the difference between the magnitude of the H-S outcome and the expected value of the high-uncertainty choice. A negative/positive difference on the abscissa indicates that the H-S outcome magnitude was greater than/less than the expected value of the high-uncertainty choice (gain/loss, respectively). A negative or positive difference on the ordinate reflects a greater likelihood to make high-uncertainty choices following H-S or H-L outcomes, respectively.

The group differences as revealed by the preceding analysis suggest that the H-S outcome may have been encoded differently (i.e., gain or loss) depending on its magnitude. However, as the magnitude of the H-S reward co-varied with the expected value of the high-uncertainty choice, it is difficult to disentangle the source of the effects. Specifically, the significant differences (or lack thereof) between H-S and H-L outcomes may reflect whether the corresponding reward magnitudes were less than or greater than the expected value of the high-uncertainty choice, respectively. If so, then the rats should show differential post H-L and post H-S choice behavior depending on how the magnitudes of these outcomes compared to the expected value of the high-uncertainty choice. Accordingly, a more sensitive analysis was warranted to better unravel the local versus global effects of reward magnitude on choice behavior. Fig. 3 shows the difference score between post H-L and post H-S choice behavior as a function of the relationship between the H-S reward magnitude and the expected value of the high-uncertainty choice; these difference scores were calculated by subtracting the log odds of high-uncertainty choices following H-S outcomes from the log odds following H-L outcomes for each probability. Positive scores indicate greater win-stay following H-L outcomes than following H-S outcomes, indicating sensitivity to high-uncertainty outcome magnitude. If the reference point for high-uncertainty gains and losses is the expected value of the high-uncertainty choice, then the difference score should approximate zero when both H-S and H-L outcomes are gains (right side of the vertical dashed line) and should be considerably greater than zero when only the H-L outcome is a gain (i.e., less high-uncertainty choice behavior following a H-S loss; left side of vertical dashed line). As seen in Fig. 3, there were no apparent effects of H-S reward magnitude as a gain or a loss relative to the expected value of the high-uncertainty choice on subsequent choice behavior. A linear regression analysis determined whether the slopes for any of the groups were different than zero, as would be produced by a high-uncertainty reference point. Of the 168 data points involved in this analysis (24 subjects × 7 expected values of high-uncertainty food), there were 31 missing data points; twenty of these 31 missing data points were due to the absence of data following either H-S or H-L outcomes at probabilities of high-uncertainty food .10 and .25, in which the number of total high-uncertainty choices (and thereby the total number of H-S and H-L outcomes) was relatively small and thus some rats did not have the opportunity to make choices following these outcomes. All but two rats (both in Group 1–11) had at least 5 data points included in the analysis. As the exclusion of these rats did not change the interpretation of the analysis, such that the statistical significance of the results was not considerably affected, these animals were not excluded from analysis. Ultimately, the analysis revealed that the slopes were not significantly different from zero.

### Discussion

The goal of the present experiment was to determine the effect of H-S outcome magnitude on global and local probabilistic choice behavior. In conjunction with previous research [21,33,39,40,54–56], an increase in the probability of receiving the probabilistic (i.e., high-uncertainty) outcome produced an increase in probabilistic choice behavior (Fig. 1A). Furthermore, increases in the magnitude of the H-S outcome increased high-uncertainty choices, and this result was not explicable by differences in the expected value of the high-uncertainty choice (Fig. 1B). According to normative/rational theories of choice behavior, the rats in Group 1–11 were behaving irrationally, avoiding the high-uncertainty choice despite the potential to earn considerably more food at higher probabilities, consistent with previous research demonstrating that human and non-human animals do not make choices in accordance with normative theories [57,58]. Furthermore, these results cannot be explained in terms of differences in satiety due to differences in reward magnitude; specifically, in accordance with the energy-budget literature suggesting that satiety should ultimately reduce risk-taking [59,60], an effect of satiety would have been evident had the rats receiving the most reward (Group 4–11) been more risk averse. As shown in Figs. 1 and 2, this was not exhibited. Thus, the group differences in choice behavior were influenced by a rather strong effect of the magnitude of the H-S reward, with the groups that received a greater H-S reward magnitude ultimately making more high-uncertainty choices. Therefore, while the rats demonstrated some sensitivity to expected value by showing a general increase in high-uncertainty choice behavior with increases in the probability of high-uncertainty food, the corresponding behavior critically depended on the individual outcomes of high-uncertainty choices.

The reward magnitude of the H-S outcome also had an effect on local choice behavior. As seen in Fig. 2, increases in H-S outcome magnitude increased high-uncertainty choice behavior following the H-S outcome. Importantly, the more targeted analyses of choice behavior following H-S and H-L outcomes revealed significant differences in high-uncertainty choice behavior in Groups 1–11 and 2–11, but not in Group 4–11. These results suggest that the 1- and 2-pellet outcomes were regarded as losses while the 4- and 11-pellet outcomes were regarded as gains. As described above, high-uncertainty gains and losses may be distinguished by whether they are greater than or equal to either zero, the expected value of the high-uncertainty outcome, or the alternative low-uncertainty outcome. Traditionally, win-stay/lose-shift behavior has been explained in terms of a high-uncertainty reference point. However, this explanation was not supported by the difference score analysis between post H-S and post H-L choice behavior as a function of high-uncertainty reward probability (see Fig. 3).

A zero-outcome reference point is consistent with the overall win-stay/lose-shift behavior seen in the local choice analysis. However, a zero-outcome reference point cannot account for the large group differences in global choice behavior (Figs. 1 and 2), as each group would have been exposed to relatively comparable gains and losses. Therefore, the significant effect of H-S outcome magnitude on global choice behavior (Fig. 1) argues against a zero-outcome reference point. However, the use of a zero-outcome reference point is tested more explicitly in Experiment 3.

Accordingly, the interesting proposition raised by the present results is that, although reward omission has been shown to be a critical determinant of subsequent behavior [33,61,62], high-uncertainty gains and losses were distinguished relative to a low-uncertainty-outcome reference point. Specifically, as the expected value of the low-uncertainty choice was constant at a value of 3, the high-uncertainty magnitudes were consistently either greater than or less than the expected value of the low-uncertainty choice. Indeed, such constancy accounts for the corresponding patterns of behavior in Fig. 3. Furthermore, assuming a low-uncertainty reference point, the reduction in probabilistic choice behavior in Groups 1–11 and 2–11 suggests that these groups may have experienced more probabilistic losses (0 and 1, 0 and 2) than Group 4–11 (0) did. However, while a zero-outcome reference point could not account for the results alone, reward omission may have produced an additional effect on behavior above and beyond the low-uncertainty reference point, at least at the level of win-stay/lose-shift behavior.

The present results also suggest that rats may be more sensitive to a relative loss than to a relative gain [16,18,63]. In all groups, the absolute difference between the magnitudes of the H-Z and H-S outcomes was smaller than that of the H-S and H-L outcomes; however, while Groups 1–11 and 2–11 showed significantly different choice behavior following H-Z, H-S, and H-L outcomes (i.e., two losses and one gain), Group 4–11 showed relatively similar choice behavior following H-S and H-L outcomes (i.e., two gains). This latter finding suggests that as long as the outcome of a successful “gamble” is greater than the low-uncertainty reference point, then outcome magnitude may have a relatively small effect on subsequent high-uncertainty choice behavior, compared to if the outcome of a gamble is smaller than the low-uncertainty reference point. Such differential sensitivity to the magnitude of the high-uncertainty outcomes, dependent on how such outcomes compare to the value of the low-uncertainty choice, may then reflect a differential sensitivity to relative gains versus relative losses [16,18,63]. One direct test of this hypothesis would be to vary H-L magnitude while maintaining its status as a gain relative to a low-uncertainty reference point, which would produce little or no effect on high-uncertainty choice behavior. Specifically, while it is hypothesized that gains and losses are not simply categorical dichotomies, it is also hypothesized that they are differentially scaled. That is, a diminished sensitivity to increasing gains is expected to produce relatively similar choice behavior. Accordingly, this question was the focus of Experiment 2.

## Experiment 2

### Method

**Animals**. Twenty-four experimentally-naïve rats, approximately 45 days of age on arrival, served as subjects. The housing and husbandry conditions were identical to Experiment 1, with the exception that the colony room lights turned off at 8 am.

**Apparatus**. The experimental apparatus was identical to Experiment 1.

### Procedure

**Magazine and lever-press training**. The magazine and lever-press training procedures were identical to Experiment 1, except that one rat required a third session of lever-press training.

**Probabilistic choice task**. Training was identical to Experiment 1 with some exceptions. The H-L reward magnitude was 6, 9, or 11 pellets in Groups 4–6, 4–9, and 4–11, respectively, while the H-S magnitude was 4 pellets in all groups. The two orders of high-uncertainty food probabilities for Phases 3–6 were .90, .33, .67, .10; and, .10, .67, .33, .90, following initial baseline training at the .50 probability (see Table 2 for expected values). In Phase 7, all rats were returned to the probability of .50. Phases 1 and 2 lasted for 20 sessions each, and Phases 3–7 lasted for 10 sessions each. Sessions terminated following the completion of 100 free choice trials or after 2 hr.

**Table 2:** Expected value of the high-uncertainty choice as a function of the probability of food for a high-uncertainty choice for each group in Experiment 2.

Probability	Group 4–6	Group 4–9	Group 4–11
.10	0.50	0.65	0.75
.33	1.65	2.17	2.50
.50	2.50	3.25	3.75
.67	3.35	4.33	5.00
.90	4.50	5.85	6.75

Group 4–6 received 4 or 6 pellets following a high-uncertainty choice, Group 4–9, 4 or 9 pellets, and Group 4–11, 4 or 11 pellets.

**Data analysis**. Data analysis was the same as in Experiment 1. Specifically, an ANOVA with factors of group (4–6, 4–9, 4–11) and order of probability exposure was conducted on choice data from Phase 2 and Phase 7 (i.e., when the probability of high-uncertainty food equaled .50). Analysis did not reveal Group × Order or Phase × Group × Order interactions, suggesting that the order of probabilities did not interact with any effect of group (i.e., an effect of H-L reward magnitude), the primary effect of interest. Further, the rats’ molar choice behavior was stable across the final five sessions of each phase. Across Phases 3–7, the mean absolute deviation in choice percentages between each of the final five sessions and the mean of the final five sessions of each phase was 5.9%.

### Results

**Fig 4:**
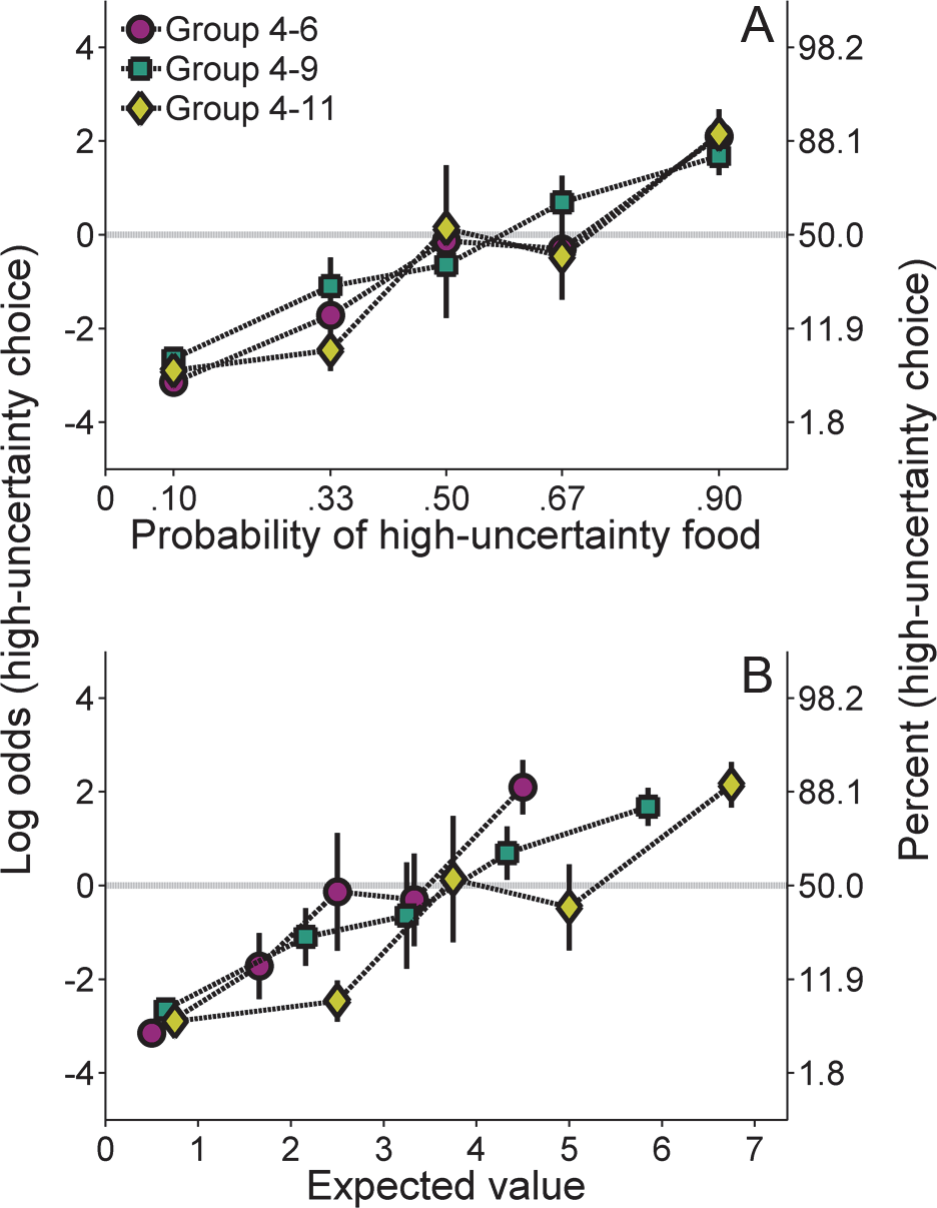
Mean (±SEM) log odds of high-uncertainty choices for each group in Experiment 2 as a function of the probability of high-uncertainty food delivery (A) and the expected value of a high-uncertainty outcome (B). A second ordinate showing percentages corresponding to the log odds values is included to aid interpretation.

**Molar analysis**. Fig. 4A shows the log odds of high-uncertainty choices as a function of the probability of high-uncertainty food delivery (Cronbach’s α = .57). All groups showed an increase in high-uncertainty choices as a function of the probability of high-uncertainty reward, but there were no systematic group differences. An ANOVA with probability as the within groups factor and group as the between groups factor revealed a main effect of probability, *F*(4, 84) = 22.64, *p* < .001, η_p_^2^ = .519. Fig. 4B shows the log odds of high-uncertainty choices as a function of the expected value of the high-uncertainty choice. While each group showed an increase in high-uncertainty choice behavior with increases in the expected value of the high-uncertainty choice, expected value did not appear to govern molar choice behavior. Instead, the functions superposed better when plotted against probability of high-uncertainty food.

**Fig 5:**
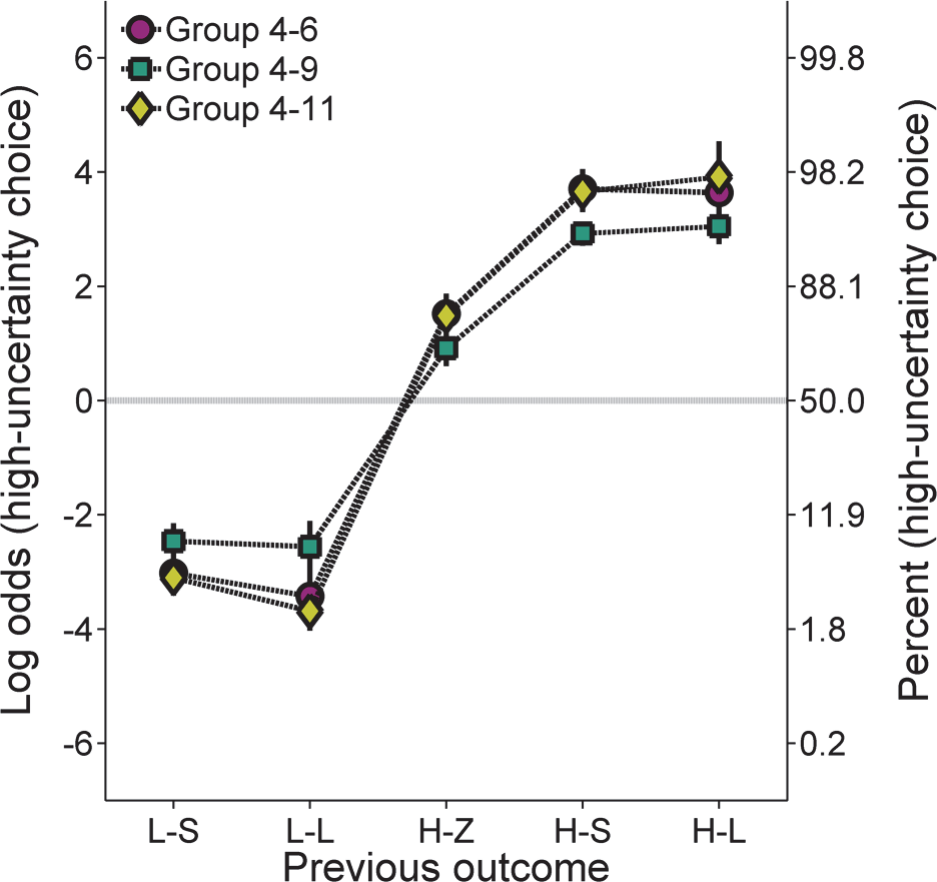
Mean (±SEM) log odds of high-uncertainty choices for each group in Experiment 2 as a function of the outcome of the previous choice, collapsed across the probability of high-uncertainty food delivery. A second ordinate showing percentages corresponding to the log odds values was included to aid interpretation. L-S = low-uncertainty-small; L-L = low-uncertainty-large; H-Z = high-uncertainty-zero; H-S = high-uncertainty-small; H-L = high-uncertainty-large.

**Molecular analysis**. Fig. 5 shows the effects of the previous outcome on subsequent choice behavior (Cronbach’s α = .51). The groups were more likely to make low-uncertainty choices following low-uncertainty outcomes, and high-uncertainty choices following high-uncertainty outcomes. Additionally, all groups showed an increased tendency to make high-uncertainty choices following H-S and H-L outcomes than following H-Z outcomes. An ANOVA with previous outcome as the within groups factor and group as the between groups factor revealed a main effect of previous outcome, *F*(4, 84) = 295.69, *p* < .001, η_P_^2^ = .934, and a Previous Outcome × Group interaction, *F*(8, 84) = 2.16, *p* = .039, η_P_^2^ = .171. To explore the Previous Outcome × Group interaction, simple effects analyses using a one-way ANOVA with group as the between groups factor were conducted on the log odds of high-uncertainty choices following each outcome to evaluate the effect of group on choice behavior following each individual outcome. These analyses did not reveal a main effect of group following any of the previous outcomes. The interaction was most likely due to the lesser degree of win-stay/lose-shift behavior in Group 4–9, but this was not localizable to any of the individual comparisons.

To address the hypothesis that rats exhibit differential sensitivities to high-uncertainty gains and losses relative to a low-uncertainty reference point, additional analyses were performed to determine if there were differences in choice behavior following H-S and H-L outcomes, as in Experiment 1. An ANOVA with previous outcome (H-S, H-L) as the within groups factor and group as the between groups factor did not reveal any significant effects.

**Fig 6:**
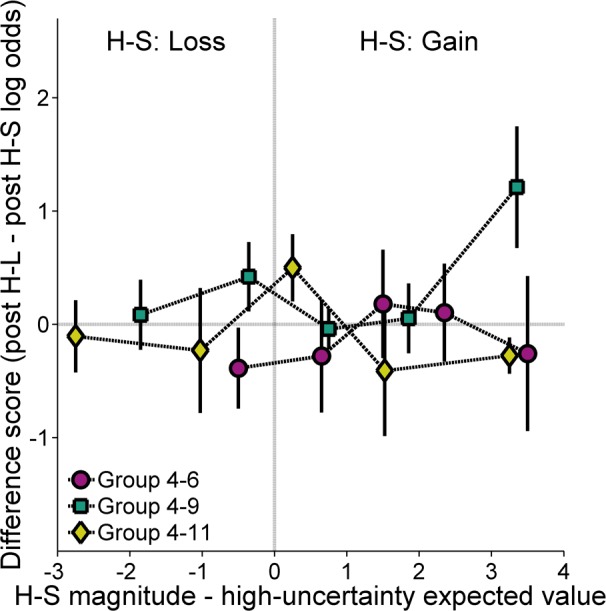
Mean (±SEM) difference score between the log odds of high-uncertainty choices following high-uncertainty-large (H-L) outcomes and the log odds of high-uncertainty choices following high-uncertainty-small (H-S) outcomes for each group in Experiment 2 as a function of the difference between the magnitude of the H-S outcome and the expected value of the high-uncertainty choice. A negative/positive difference on the abscissa indicates that the H-S outcome magnitude was greater than/less than the expected value of the high-uncertainty choice (gain/loss, respectively). A negative or positive difference on the ordinate reflects a greater likelihood to make high-uncertainty choices following H-S or H-L outcomes, respectively.

To further test the effect of H-S and H-L outcomes on subsequent choice behavior, in relation to a high-uncertainty reference point, Fig. 6 shows the difference between choice behavior following H-L and H-S outcomes as a function of the difference between the expected value of the high-uncertainty choice and the magnitude of the H-S outcome. Positive difference scores reflect greater high-uncertainty choices following the larger magnitude (H-L) outcome than following the H-S outcome. The tendency for the functions in Fig. 6 to approximate a zero slope suggests that there was little, if any, difference in choice behavior following the high-uncertainty food outcomes across conditions in which the H-S outcome was greater or less than the expected value of the high-uncertainty choice. The data were subjected to a linear regression analysis to determine whether the functional slopes for any of the groups were different than zero, as would be expected assuming a high-uncertainty reference point. Of the 120 data points involved in this analysis (24 subjects × 5 expected values of high-uncertainty food), there were 16 missing data points; thirteen of these 16 missing data points were due to the absence of data following either H-S or H-L outcomes at probabilities of high-uncertainty food .1, in which the number of total high-uncertainty choices (and thereby the total number of H-S and H-L outcomes) was relatively small. All but two rats had at least 4 data points included in the analysis. As the exclusion of these rats did not change the interpretation of the analysis, such that the statistical significance of the results was not considerably affected, these animals were not excluded from analysis. Ultimately, the analysis revealed that the slopes were not significantly different from zero.

### Discussion

The goal of the present experiment was to determine the effect of H-L outcome magnitude on global and local probabilistic choice behavior. Similar to Experiment 1, the increase in the probability of high-uncertainty food delivery produced a general increase in high-uncertainty choices (Fig. 4A). However, in contrast to the effects of different H-S reward magnitudes on choice behavior (Experiment 1), the changes in H-L magnitude did not affect probabilistic choice behavior. This pattern was not altered when the choice functions were plotted relative to the expected value of the high-uncertainty choice (Fig. 4B).

The present results, in conjunction with those of Experiment 1, cannot be explained as being due to differences in the expected value of the high-uncertainty choice. The parameters used in the current experiment were designed to produce expected values that matched or closely approximated those in Experiment 1. Groups 4–11 were identical in the two experiments, Group 2–11 matched the expected values of Group 4–9, and Group 1–11 closely approximated the expected values of Group 4–6 (see Tables 1 and 2). Despite the similar expected values, the effects of the magnitude manipulations in the two experiments were decidedly different, lending further support to the notion that the rats used a low-uncertainty reference point to gauge high-uncertainty gains and losses and that the rats were more sensitive to losses relative to that reference point. There are two possible explanations of the results: (1) rats may be unable to discriminate larger outcome magnitudes as readily as smaller outcome magnitudes, consistent with Weber’s law; and (2) the rats may experience diminishing marginal utility of increasing gains, ultimately resulting in reduced sensitivities to larger gains. The present results cannot differentiate these two possibilities, and they may indeed be related. In either event, the findings suggest that further consideration of non-linear scaling of magnitudes be undertaken (see General Discussion).

The different H-L reward magnitudes also did not affect local choice behavior. The H-Z outcomes appeared to be encoded as losses, given the reduction in subsequent high-uncertainty choice behavior following H-Z outcomes, whereas the high-uncertainty food outcomes appeared to be encoded as gains of similar value. Therefore, high-uncertainty gains and losses do not seem to have been gauged relative to the expected value of the high-uncertainty choice. However, the present data do not conclusively eliminate a role for a zero-outcome reference point, as the reduced sensitivity to gains could be explained by the non-zero high-uncertainty food outcomes being greater than both the low-uncertainty reference point and a zero-outcome reference point. Accordingly, a more thorough elucidation of low-uncertainty versus zero-outcome reference point use will be examined in Experiment 3.

The current hypothesis that high-uncertainty food rewards serve as losses and gains relative to a low-uncertainty reference point is critical as it is inconsistent with many theories of decision making (see the General Discussion for further details). However, it is possible that the one-pellet outcome in Experiment 1 was regarded as a loss because it was subjectively closer to the zero-pellet outcome than it was to the 11-pellet outcome (gain). To assess sensitivity to the 0- and 1-pellet outcomes, Experiment 3 delivered within groups manipulations of probability of receipt of each of these outcomes. If rats are more sensitive to 0-pellet outcomes, then changing the probability of the 0-pellet outcome should produce a greater change in high-uncertainty choices than changing the probability of the 1-pellet outcome. In addition, the rats received within groups manipulations of low-uncertainty outcome magnitude across phases to provide further indication of loss assessment of the 0- and 1-pellet outcomes.

Lastly, it is also possible that the individual values of the low-uncertainty choice comprised the reference point instead of the corresponding expected value. Specifically, the 1-pellet uncertain outcome in Group 1–11 (Experiment 1) may have been regarded as a loss because it was less than both the 2- and 4-pellet low-uncertainty outcomes, and the 2-pellet high-uncertainty outcome for Group 2–11 may have been regarded as a loss (but less of a loss) because it was less than only the 4-pellet low-uncertainty outcome. This question was addressed in Experiment 3 by maintaining a constant expected value of the low-uncertainty choice and employing a between groups manipulation of the individual low-uncertainty outcome values.

## Experiment 3

### Method

**Animals**. Twenty-four experimentally-naïve rats, approximately 45 days of age on arrival, served as subjects. The housing and husbandry conditions were identical to Experiment 2.

**Apparatus**. The experimental apparatus was identical to Experiments 1 and 2.

### Procedure

**Magazine and lever-press training**. The magazine and lever-press training procedures were identical to Experiments 1 and 2. Magazine training lasted for one session and lever-press training lasted for three sessions.

**Experiment 3A: High-uncertainty probability training**. The high-uncertainty probability training procedure was identical to that of Experiment 1 with some exceptions. The rats were randomly partitioned into Group 2–4 and Group 1–5, in which the group names refer to the L-S and L-L outcomes. The probability of each low-uncertainty outcome was .50 to equate overall expected value in the two groups at 3 pellets. For both groups, a high-uncertainty choice resulted in the probabilistic delivery of 0, 1, or 11 pellets. The within groups manipulation was the probability of the 0-pellet or 1-pellet outcomes (see Table 3). For half of the rats in each group, the first set of phases involved manipulation of the 0-pellet outcome. In Phase 1, the probability of 0 pellets, p[0], was .1, so that the probability of receiving 1 or 11 pellets was .9 (p[1] = .45 and p[11] = .45). In Phases 2–4, p[0] was .9, .5, and .1, respectively, and p[1] and p[11] were each equal to .05, .25, and .45, respectively. For the other half of the rats in each group, the first set of phases involved manipulations of the 1-pellet outcome, so that in Phases 1–4, p[1] was equal to .1, .9, .5, and .1, respectively, and p[0] and p[11] were each equal to .45, .05, .25, and .45, respectively. In the second set of phases, those rats first experiencing the p[0] manipulation then experienced the p[1] manipulation and vice versa. In both sets of phases, the outcome that followed the four high-uncertainty forced choice trials was either the 11-pellet outcome or the other high-uncertainty outcome with the same probability of delivery (i.e., 0 pellets in the p[1] condition, 1 pellet in the P[0] condition). Phase 1 lasted for 20 sessions. Phase 2 lasted for 10 sessions. Phases 3–5 each lasted for 11 sessions. Phases 6–7 each lasted for 10 sessions.

**Table 3:** Expected value of the high-uncertainty choice as a function of the probability of receiving 0 pellets (P[0]) or 1 pellet (P[1]) following a high-uncertainty choice in the high-uncertainty probability training procedure of Experiment 3A.

Probability	P[0]		p[1]
.10	5.40		5.05
.50	3.00		3.25
.90	0.60		1.45

**Experiment 3B: Low-uncertainty magnitude training**. The training procedure was identical to high-uncertainty probability training with a few exceptions. Low-uncertainty choices resulted in the delivery of only one outcome magnitude, which was 3, 1, 5, and 3 pellets or 3, 5, 1, and 3 pellets in Phases 1–4, respectively. The high-uncertainty choice outcomes were 0, 1, and 11 pellets, and the probability of receiving 1 or 11 pellets following a high-uncertainty choice was .25 across phases (i.e., p[food] = .50) with p[0] equal to .5. Phase 1 lasted for 11 sessions. Phases 2–4 lasted for 10 sessions.

**Data analysis**. Data analysis was the same as in Experiments 1 and 2. For analysis of high-uncertainty probability training, the final five sessions of Phases 2–7 were used for data analyses. Due to an equipment error that resulted in data loss, sessions 6, 7, 8, 10, and 11 of Phase 3 were used for analysis of one rat, and sessions 6, 7, 9, 10, and 11 of Phase 5 were used for analysis of four rats. The rats’ molar choice behavior was stable across the final five sessions of each phase. Across Phases 2–7, the mean absolute deviation in choice percentages between each of the final five sessions and the mean of the final five sessions of each phase was 3.5%. Three rats were removed from the molecular analyses of the high-uncertainty probability training phase due to missing data. Specifically, of the 240 data points involved in this analysis (24 subjects × 5 previous outcomes × 2 probability conditions), there were 3 missing data points, one per each of the rats removed from this analysis. Two rats did not receive any H-S outcomes and one rat did not receive any H-L outcomes in the P[0] condition, such that there were not any data as a function of these previous outcomes. The missing data occurred because of general risk aversion and relatively small number of high-uncertainty choices in those individuals. For low-uncertainty magnitude training, the final five sessions of Phases 2–4 were used for data analyses. The rats’ molar choice behavior was stable across the final five sessions of each phase. Across Phases 2–4, the mean absolute deviation in choice percentages between each of the final five sessions and the mean of the final five sessions of each phase was 5.5%. Seven rats were removed from the molecular analyses of the low-uncertainty magnitude training phases due to missing data, leaving a total of 17 rats in this analysis. Of the 288 data points involved in this analysis (24 subjects × 4 previous outcomes × 3 low-uncertainty magnitudes), there were 12 missing data points. Half of these missing data points were due to the absence of data following a high-uncertainty magnitude when the low-uncertainty magnitude was equal to 5 pellets (i.e., when the rats were less likely to make high-uncertainty choices). With the exception of one rat that was missing four data points due to general risk aversion, the maximum number of missing data points per rat was two.

### Results

**Fig 7:**
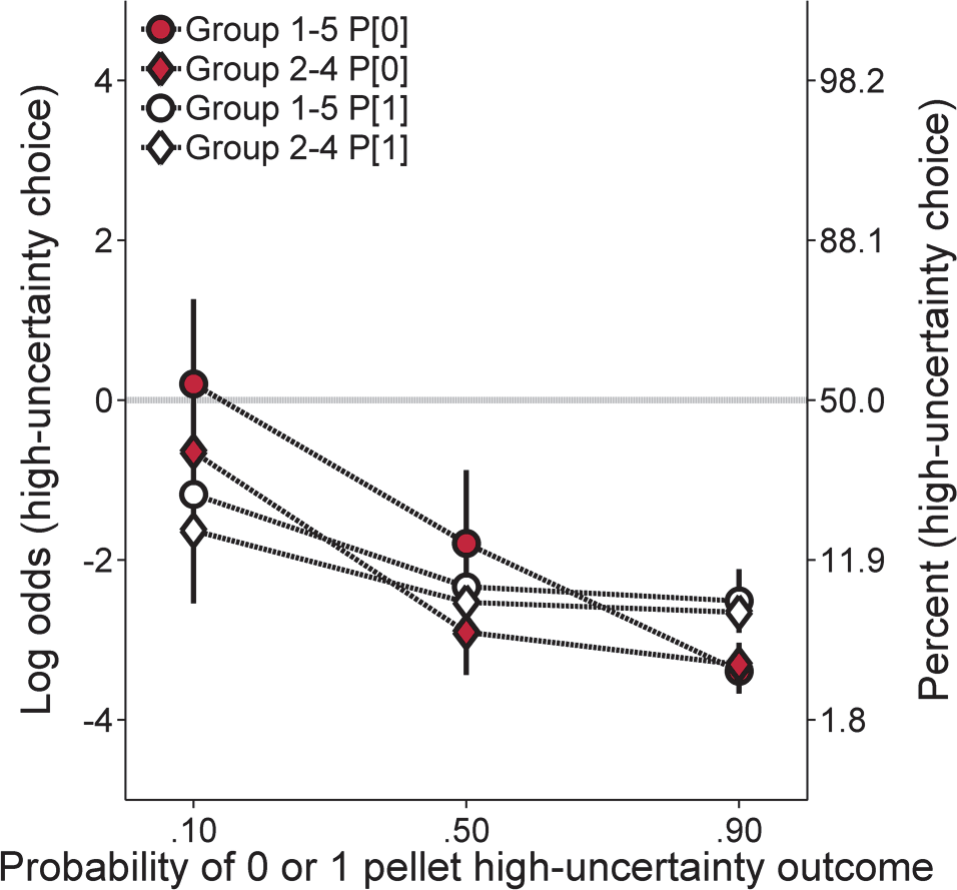
Mean (±SEM) log odds of high-uncertainty choices for each group in the high-uncertainty probability training procedure of Experiment 3A as a function of the probability of the 0-pellet outcome (P[0]) or the 1-pellet outcome (P[1]). Note that high-uncertainty choices are plotted against the probability of the manipulated outcome rather than as a function of overall probability of food, as was the case in previous figures. A second ordinate showing percentages corresponding to the log odds values was included to aid interpretation.

**Fig 8:**
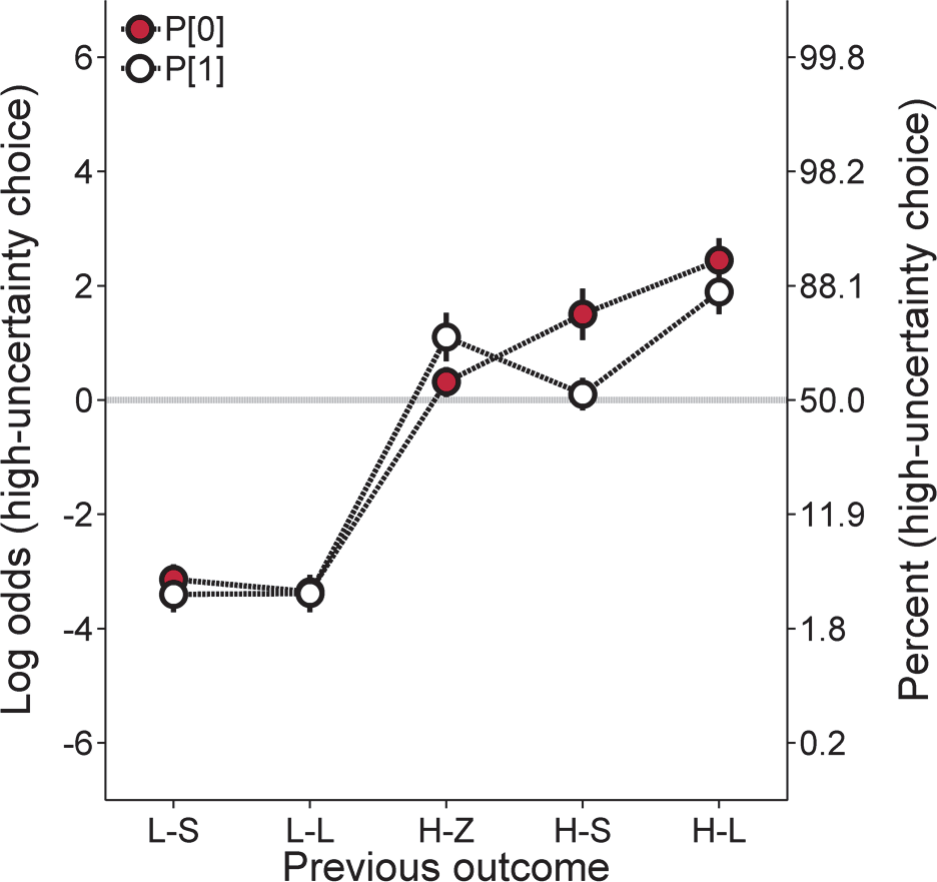
Mean (±SEM) log odds of high-uncertainty choices collapsed across groups in the high-uncertainty probability training procedure of Experiment 3A as a function of the outcome of the previous choice, collapsed across probability. A second ordinate showing percentages corresponding to the log odds values was included to aid interpretation. L-S = low-uncertainty-small; L-L = low-uncertainty-large; H-Z = high-uncertainty-zero; H-S = high-uncertainty-small; H-L = high-uncertainty-large; P[0] = probability of 0 pellets; p[1] = probability of 1 pellet.

**Experiment 3A: High-uncertainty probability training. *Molar analysis*:** Fig. 7 shows the log odds of high-uncertainty choices as a function of the probability of receiving 0 pellets (P[0]) or 1 pellet (p[1]) for a high-uncertainty choice for the two groups of rats (Cronbach’s α = .85). There was a general decrease in high-uncertainty choices as the probability of receiving the 0- or 1-pellet outcome increased, indicating that the rats were sensitive to the increased likelihood of both outcomes. Overall, the functions were generally similar for the four conditions, but the choice functions were slightly steeper for the P[0] conditions than the p[1] conditions. An ANOVA was conducted with group (2–4 and 1–5) as the between groups factor, and probability (.1, .5, and .9) and condition (P[0] and p[1]) as within groups factors. The analysis revealed a main effect of probability, *F*(2, 44) = 14.02, *p* < .001, η_p_^2^ = .389, and a Condition × Probability interaction, *F*(2, 44) = 5.95, *p* = .005, η_p_^2^ = .213. Simple effects analyses (i.e., paired samples *t*-tests) revealed significantly lower high-uncertainty choices in the P[0] condition compared to the p[1] condition at the probability of. 9, but no significant differences at other probabilities. ***Molecular analysis*:** Fig. 8 shows the log odds of high-uncertainty choices following each of the previous outcomes, collapsed across probability (Cronbach’s α = .84). The data were also collapsed across group to ease interpretation of the data and as there was no effect of group in the molar analyses. As seen in Fig. 8, the rats were generally more likely to make low-uncertainty choices after low-uncertainty outcomes (L-S, L-L) and high-uncertainty choices after high-uncertainty outcomes (H-Z, H-S, H-L). Interestingly, high-uncertainty choice behavior following H-Z and H-S outcomes depended on the probability condition (P[0] vs. p[1]). In the P[0] condition, when the probability of the H-Z outcome was manipulated such that the probabilities for the H-S and H-L outcomes were identical, rats were less likely to make high-uncertainty choices following H-Z outcomes than following H-S outcomes. However, in the p[1] condition, when the probability of the H-S outcome was manipulated such that the probabilities for the H-Z and H-L outcomes were identical, the rats displayed fewer high-uncertainty choices following H-S outcomes than following H-Z outcomes. There were no apparent differences following H-L outcomes. An ANOVA with previous outcome and condition as within groups factors and group as the between groups factor revealed a main effect of previous outcome, *F*(4, 76) = 114.92, *p* < .001, η_p_^2^ = .858, and a Condition × Previous Outcome interaction, *F*(4, 76) = 10.06, *p* < .001, η_p_^2^ = .346. Paired samples *t*-tests conducted on the interaction disclosed significantly fewer high-uncertainty choices following H-Z outcomes in the P[0] condition than in the p[1] condition, and significantly fewer high-uncertainty choices following H-S outcomes in the p[1] condition than in the P[0] condition.

**Fig 9:**
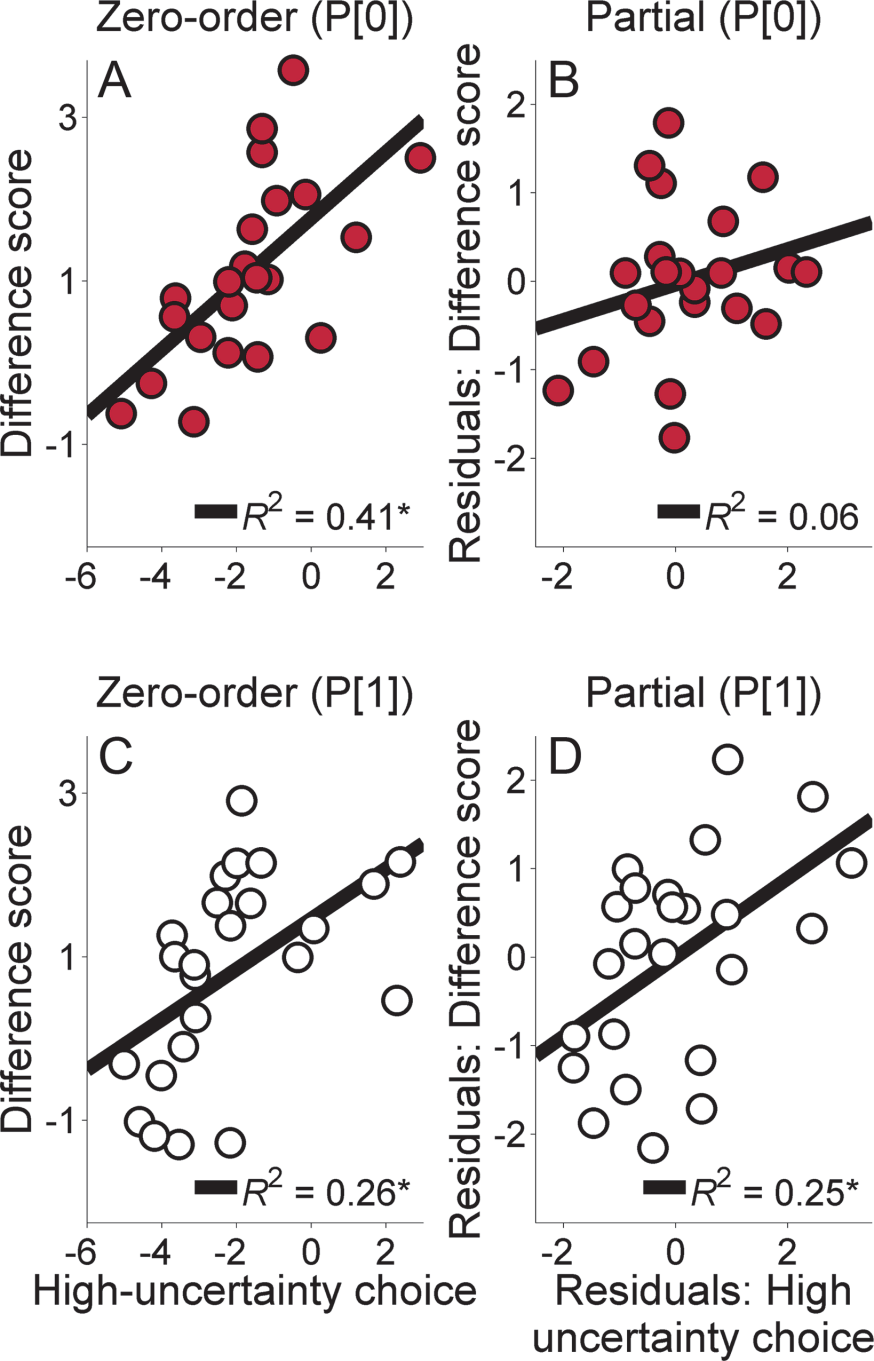
A: Zero-order correlation between the mean log odds of high-uncertainty choices across probabilities of the P[0] condition (abscissa) and the difference score in the log odds of high-uncertainty choices following high-uncertainty-zero (H-Z) outcomes relative to high-uncertainty-small (H-S) outcomes (ordinate). Here, post H-Z high-uncertainty choice behavior was subtracted from post H-S high-uncertainty choice behavior. B: Partial correlation between the two variables in Panel A, controlling for mean post H-Z choice behavior. The abscissa and ordinate show the residuals derived from the correlations of these variables with mean post H-Z choice behavior. C: Zero-order correlation between the mean log odds of high-uncertainty choices across probabilities of the p[1] condition (abscissa) and the difference score in the log odds of high-uncertainty choices following H-S outcomes relative to H-Z outcomes (ordinate). Here, post H-S high-uncertainty choice behavior was subtracted from post H-Z high-uncertainty choice behavior. D: Partial correlation between these two variables, controlling for mean post H-S choice behavior. The abscissa and ordinate show the residuals derived from the correlations of these variables with mean post H-S choice behavior. The best fitting regression line and variance accounted for (R^2^) is shown in each panel.

Within a win-stay/lose-shift framework, the present results suggest that when the probability of receiving 1 pellet was manipulated, the reception of 1 pellet was regarded as a greater loss than the reception of 0 pellets; the reverse was true in the P[0] condition. While this pattern seems counterintuitive given that 0 is objectively and numerically smaller than 1, these results may reflect different underlying mechanisms. Recall that in the p[1] condition, the initial high-uncertainty forced choice trials terminated in either 0 or 11 pellets. This procedure is comparable to gambling and probabilistic choice paradigms in which high-uncertainty choices result in either a large gain or no reward [64]. Given such an analogous structure, an additional analysis was conducted to determine if the decrease in post-outcome behavior was related to an individual rat’s propensity to make high-uncertainty choices. A partial correlational analysis was conducted between the rats’ mean log odds of high-uncertainty choices (collapsed across probability) in the P[0] and p[1] conditions (collapsing across groups) and the reduction in the log odds of high-uncertainty choices following H-S outcomes compared to H-Z outcomes (H-S – H-Z for P[0] and H-Z – H-S for p[1]), controlling for overall post H-Z or H-S choice behavior. Two animals were excluded from the analysis of the P[0] condition due to missing data. Specifically, as described above, two animals did not receive the H-S outcome in the P[0] condition, such that there were no choice data as a function of this previous outcome. The missing data occurred because of general risk aversion and relatively small number of high-uncertainty choices in those individuals. Specifically, in Fig. 9A, the difference score refers to post H-S high-uncertainty choice behavior minus post H-Z high-uncertainty choice behavior, such that a larger difference reflects more high-uncertainty choice behavior following H-S outcomes. In Fig. 9C, the difference score is reversed; that is, the difference score refers to post H-Z high-uncertainty choice behavior minus post H-S high-uncertainty choice behavior, so that a larger difference here reflects more high-uncertainty choice behavior following H-Z outcomes. These difference score values are reversed due to the opposing directions of these data in Fig. 8. The analysis of the P[0] conditions revealed a significant zero-order correlation between mean log odds of high-uncertainty choices and the change in high-uncertainty choice behavior following H-Z outcomes relative to H-S outcomes, *r* = .64, *p* = .001 (Fig. 9A), but the partial correlation was not significant when controlling for mean post H-Z choice behavior, *r* = .26, *p* = .265 (Fig. 9B). For the p[1] conditions, there was a significant positive zero-order correlation, *r* = .51, *p* = .010, and a significant positive partial correlation, *r* = .50, *p* = .015, controlling for mean post H-S choice behavior (Fig. 9C and 9D).

**Fig 10:**
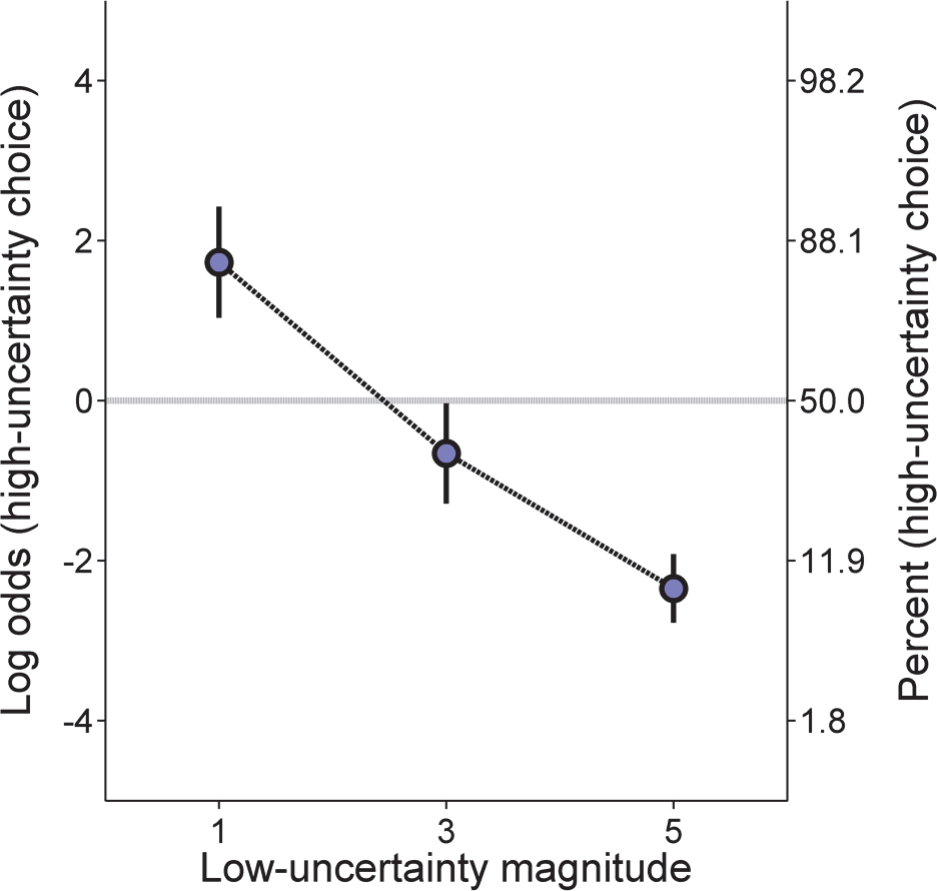
Mean (±SEM) log odds of high-uncertainty choices in the low-uncertainty magnitude training procedure of Experiment 3B as a function of the outcome magnitude of a low-uncertainty choice. A second ordinate showing percentages corresponding to the log odds values is included to aid interpretation.

**Fig 11:**
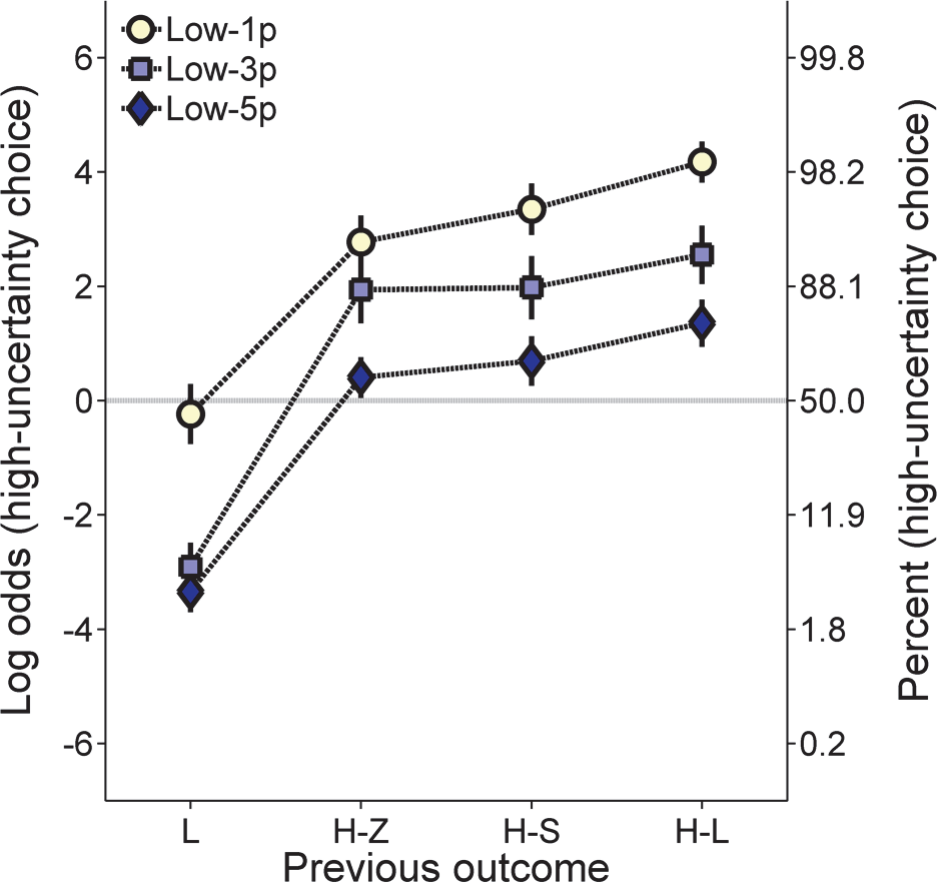
Mean (±SEM) log odds of high-uncertainty choices in the low-uncertainty magnitude training procedure of Experiment 3B as a function of the outcome of the previous choice and the outcome magnitude (pellets, *p*) of a low-uncertainty outcome choice. A second ordinate showing percentages corresponding to the log odds values is included to aid interpretation.

**Experiment 3B: Low-certainty magnitude training. *Molar analysis*:** Fig. 10 shows the log odds of high-uncertainty choices as a function of the low-uncertainty outcome magnitude (Cronbach’s α = .84). The rats made significantly fewer high-uncertainty choices with increases in the low-uncertainty reward magnitude, *F*(2, 46) = 32.01, *p* < .001, η_p_^2^ = .582. ***Molecular analysis*:** Fig. 11 shows the log odds of high-uncertainty choices as a function of the previous outcome and the low-uncertainty outcome magnitude (Cronbach’s α = .91). The rats made more low-uncertainty choices following low-uncertainty outcomes and more high-uncertainty choices following high-uncertainty outcomes. An ANOVA with low-uncertainty outcome magnitude and previous outcome as within groups factors revealed a main effect of low-uncertainty outcome magnitude, *F*(2, 32) = 38.30, *p* < .001, η_p_^2^ = .705, a main effect of previous outcome, *F*(3, 48) = 86.00, *p* < .001, η_p_^2^ = .843, and a Low-Uncertainty Outcome Magnitude × Previous Outcome interaction, *F*(6, 96) = 2.70, *p* = .018, η_p_^2^ = .144. Paired-sample *t*-tests were conducted on post H-Z and post H-S choice behavior to explore the interaction. When the low-uncertainty outcome was 1 pellet, the rats were significantly more likely to make high-uncertainty choices following the H-S outcome than following the H-Z outcome, *t*(16) = 2.28, *p* = .037. However, when the low-uncertainty outcome was 3 or 5 pellets, the rats did not differ in their choices following H-S and H-Z outcomes.

### Discussion

The goal of the current experiment was to further investigate the hypothesis that the expected value of the low-uncertainty choice served as the reference point for distinguishing high-uncertainty gains from losses. During high-uncertainty probability training, the rats showed an increase in high-uncertainty choice behavior as a function of the probability of the H-L outcome, similar to results shown in Experiments 1 and 2. Interestingly, this effect of H-L outcome probability was moderated by condition (Fig. 7); the P[0] condition produced steeper choice functions than the p[1] condition. Therefore, these results generally corroborate the results of Experiments 1 and 2, in that the rats were more sensitive to zero than non-zero losses. However, the rats were sensitive to the p[1] changes, indicating that they treated the 1-pellet outcome as a loss. Furthermore, the absence of group effects on molar choice behavior suggests that the expected value of the low-uncertainty choice governed choice behavior to a greater degree than the individual magnitudes. Thus, the general risk aversion observed in Fig. 7 was likely induced by the probabilistic delivery of two losses (H-Z, H-S) versus one gain (H-L). Indeed, the rats were risk averse when the expected value of the high-uncertainty choice approximated that of the low-uncertainty choice (at a probability of .5), suggesting that: (1) the likelihood of a loss outweighed the possibility of an 11-pellet gain, and (2) the expected value of the high-uncertainty choice influenced choice behavior to a lesser degree than did the possibility of receiving a high-uncertainty loss.

The results from the molar analyses were complemented by comparable results at the molecular level. Similar to Experiments 1 and 2, the rats made more low-uncertainty choices following low-uncertainty outcomes and more high-uncertainty choices following high-uncertainty outcomes [33]. The absence of a group effect on local choice behavior supported the importance of the expected value of the low-uncertainty choice, in contrast to its individual values, in governing probabilistic choice (Fig. 8). The most striking result of the current experiment was the effect of probability condition on choice behavior following H-S and H-Z outcomes (Fig. 8). In the P[1] condition, the rats were more likely to make a high-uncertainty choice following H-Z outcomes than following H-S outcomes; given that independent trial outcomes were orthogonal, these data cannot be explained by the delivery of H-Z outcomes predicting an increased likelihood of receiving food for a subsequent high-uncertainty choice. In addition, this behavioral pattern was related to the rats’ overall risk proneness in the p[1] condition, but not in the P[0] condition (Fig. 9). Specifically, in the p[1] condition, the rats that showed the greatest reduction in high-uncertainty choice behavior following H-S than following H-Z outcomes (controlling for overall H-S choices) were those that were more likely to make high-uncertainty choices at a molar level. While it may be argued that rats that do not make many high-uncertainty choices would be less affected by different high-uncertainty outcomes, the present results maintain their translational significance in showing the involvement of differential loss processing in overall risky choice behavior. Specifically, the partial correlational analyses (Fig. 9B, 9D) controlled for baseline levels of post-outcome choice behavior, therefore suggesting that the molecular processing of differential losses itself correlates with molar choice behavior. Thus, these results ultimately suggest that non-zero losses could potentially be employed in behavioral interventions to reduce baseline tendencies to make risky choices. Intuitively, this technique may convey the futility of gambling if the winning outcome is less than what could have been earned for making an alternative choice. In addition, it may be more difficult to ignore a non-zero outcome than a zero-outcome, because zero outcomes can be construed as the absence of an event occurrence.

Ultimately, as the delivery of a 1-pellet outcome resulted in a subsequent reduction rather than increase in high-uncertainty choice behavior, the present results lend direct support to the idea that uncertain outcomes in the present experiment were distinguished as gains and losses relative to the expected value of the low-uncertainty choice. The results of the low-uncertainty magnitude training phases corroborated this explanation, as global choice behavior was substantially affected by the objective value of the low-uncertainty outcome (Fig. 10). Specifically, even though there was the same absolute relationship between the H-Z and H-S outcomes across phases, there was a difference in local high-uncertainty choice behavior depending on the low-uncertainty outcome magnitude; this supports the present hypotheses of a low-uncertainty reference point. If high-uncertainty gains and losses (and corresponding win-stay/lose-shift behavior) were distinguished purely with respect to the expected value of the high-uncertainty choice, then high-uncertainty choice behavior following each of the high-uncertainty outcomes should have been relatively constant as a function of low-uncertainty outcome magnitude (Fig. 11), which was not the case here. While such results may appear inconsistent with the above explanations of greater sensitivity to greater losses, it is important to remember that a diminishing sensitivity with increased separation from the reference point is a core tenet of theoretical accounts of decision-making [16], an issue that is considered in more detail in the General Discussion. If both the H-S and H-Z outcomes were losses, then increased separation from the 5-pellet low-uncertainty reward may be assumed to produce a more similar treatment of the loss outcomes. Therefore, the changes in post H-Z and post H-S behavior as a function of the low-uncertainty outcome magnitude suggests that the 1-pellet H-S outcome was regarded as a loss because of its relationship to the low-uncertainty choice expected value rather than its proximity to 0. Accordingly, the present results corroborate those of the experiments above, suggesting that rats’ evaluative mechanisms involve comparison to outcomes that could have been obtained had a different choice been made.

The current results, especially the effect of the 1-pellet outcome, serve as an intriguing complement to more recent research that has identified “losses-disguised-as-wins” as a potentially relevant factor driving the onset of maladaptive gambling behavior. Dixon et al. [65] described a considerable proportion of gambling outcomes as positive monetary values that are objectively less than the amount wagered, making these outcomes losses, even though they are presented as wins. In other words, individuals experience losses that are presented as if they won. Dixon et al. [65] measured changes in heart rate and skin conductance responses following wins, losses, and losses-disguised-as-wins. Interestingly, while heart rate changed in seemingly similar ways following losses and losses-disguised-as-wins, skin conductance responses were more similar following wins and losses-disguised-as-wins. Thus, such results suggest that the presentation of outcomes, which may be subjectively regarded as wins or losses, elicit a complex biological response that may consequently distinguish those individuals susceptible to problematic risky decision making behaviors.

These results are especially relevant to the present experiments, as the 1-pellet outcomes in Experiments 1, 3A, and 3B may be viewed as losses-disguised-as-wins; they were less than the expected value of the low-uncertainty choice (loss) but were also positive food amounts delivered in the same way as the larger 11-pellet outcomes (win). Given the individual differences in processing the 0- and 1-pellet losses (Fig. 9), it is possible that losses-disguised-as-wins may be perceived as wins in some individuals and losses in others. As Dixon et al. [65] suggested that losses-disguised-as-wins may lead to elevated gambling behavior, individual differences in the processing of small losses may ultimately predict overall risk-taking behavior (Fig. 9). Furthermore, Dixon et al. [65] noted that wins and losses-disguised-as-wins are delivered with approximately equal percentages in some slot machine games. In the present experiment, when the 11- and 1-pellet outcomes were delivered with similar probabilities (P[0] condition), rats were more likely to make risky choices following 1-pellet outcomes than following zero-pellet outcomes, a comparable result to the losses-disguised-as-wins phenomenon. However, this was not the case when the 1-pellet outcomes were delivered with different probabilities compared to the 11-pellet outcome (p[1] condition). Thus, if the losses-disguised-as-wins phenomenon can be explained by the frequencies of differential losses, then the present results serve as a critical advancement in our understanding of the mechanisms that govern risk-taking behavior. Therefore, individual differences in decision making following different outcomes, especially losses, may in fact serve as the ultimate predictor for the likelihood of onset of problem-gambling and other maladaptive behaviors.

## General Discussion

The primary goal of the present experiments was to differentiate between three possible reference points that may affect judgments of high-uncertainty gains versus losses in rats: high-uncertainty, zero-outcome, and low-uncertainty. The collective results suggest that the rats used the expected value of the low-uncertainty outcome as a reference point for gauging high-uncertainty gains and losses (see Table 4 for an integrated summary of the results). Specifically, the rats showed significant differences in choice behavior following high-uncertainty outcomes greater than and less than the expected value of the low-uncertainty choice (Figs. 2, 5, 8 and 11). In addition, they were insensitive to the individual values that comprised the low-uncertainty outcome (Fig. 7), but showed strong sensitivity to the low-uncertainty outcome when that value was manipulated in Experiment 3 (Fig. 10). The combined results indicate that the rats were likely using the low-uncertainty expected value. However, there were no systematic differences in choice behavior following the H-S outcome depending on whether it was greater than or less than the expected value of the high-uncertainty choice (Figs. 3 and 6). This latter result suggests that the H-S outcome was not differentiated as a gain or a loss relative to a high-uncertainty reference point, as would have been suggested had there been significant differences in post H-S choice behavior depending on its gain/loss dichotomization relative to the expected value of the high-uncertainty choice.

**Table 4:** Summary of the primary results from Experiments 1–3.

	Molar Results	Molecular Results: Post High-Uncertainty-Outcome Behavior	Conclusions
Expt. 1	-Group 1–11 < 2–11 < 4–11	-Group 1–11: H-Z < H-S < H-L	-Low-uncertainty reference point
		-Group 2–11: H-Z < H-S < H-L	-Greater sensitivity to differential losses
		-Group 4–11: H-Z < H-S ≈ H-L	
Expt. 2	-Group 4–6 ≈ 4–9 ≈ 4–11	-Group 4–6: H-Z < H-S ≈ H-L	-Low-uncertainty or zero-outcome reference point
		-Group 4–9: H-Z < H-S ≈ H-L	-Reduced sensitivity to differential gains
		-Group 4–11: H-Z < H-S ≈ H-L	
Expt. 3: High-uncertainty Probability	-Group 1–5 ≈ 2–4	-Group 1–5 ≈ Group 2–4	-Low-uncertainty reference point
	-P[0] condition produced steeper choice functions than p[1] condition	-P[0] condition: H-Z < H-S	-Greater sensitivity to 0-pellet outcome
		-p[1] condition: H-Z > H-S	-Sensitivity to 1-pellet loss predicted overall high-uncertainty choices
Expt. 3: Low-uncertainty Magnitude	-Reduction in high-uncertainty choice with increases in low-uncertainty-outcome magnitude	-Reduction in high-uncertainty choice with increases in low-uncertainty outcome magnitude	-Low-uncertainty reference point
		-Differential effect of low-uncertainty magnitude on post H-Z and post H-S choice	

*Note: H-Z = high-uncertainty-zero; H-S = high-uncertainty-small; H-L = high-uncertainty-large.

In conjunction with the results suggesting a low-uncertainty reference point, a second relatively novel finding of the present experiments was that rats were more sensitive to differential losses than they were to differential gains, as has been described previously in humans [16]. Interestingly, while a recent report by Bhatti et al. [26] did postulate a possible effect of loss magnitude on loss aversion in rats, the present report included experimental manipulations that directly investigated loss versus gain sensitivity in rats. Therefore, while previous research has described the non-linear relationship between objective and subjective stimulus magnitudes in animals (i.e., Weber’s law) [66], this is the first comprehensive evidence, to our knowledge, describing differential sensitivity to gains and losses in rats within a probabilistic choice task. Furthermore, as common computational mechanisms of valuation have been assumed to follow a linear value-updating mechanism, suggesting that individuals are equally sensitive to gains and losses [67], previous computational modeling of non-human primate choice behavior has considered separate parameters for value updating in the gain and loss domain [68,69]. The present results support the use of the latter modeling approach. Therefore, as the present experiment employed multiple parametric manipulations of magnitude, in conjunction with thorough analyses of probabilistic choice behavior, the current results suggest that, like humans, rats may be differentially sensitive to probabilistic gains and losses. Altogether, these results strengthen the premise that rats serve as a viable animal model of risky decision making behaviors [15]. Indeed, in relation to this supposition, the rats showed consistent and stable behavior when tested with different choice parameters, with alpha values in the moderate to high range in all cases. This further verifies the validity of the rat as a model by demonstrating that probabilistic choice is a stable trait in rats.

### Theories of Probabilistic Choice Behavior

Several theoretical frameworks have incorporated reference points in evaluating choice outcomes as gains and losses. While these frameworks have been frequently shown to account for molar choice behavior, a more critical assessment would include their ability to account for choice behavior on a molecular scale [70,71]. Two of the most influential theories of choice behavior are prospect theory [16,18] and optimal foraging theory [43]. In prospect theory, an individual’s subjective reference point reflects what an individual currently has or rather what an individual expects/aspires to have [16]. Prospect theory [16, p. 287] predicts greater risk-seeking following losses that have not yet been adapted to [18,42,72,73]. In optimal foraging theory, choice behavior is driven by the goal to maximize energy intake at the expense of the energy needed during the act of foraging [43,59,74,75]. If a previous outcome attenuates the ability to reach this energy intake threshold, then the animal may exhibit subsequent risk seeking behavior to compensate for the prior loss [76]. Therefore, optimal foraging theory can also predict risk seeking following losses. In contrast, the results of the current experiments suggest that the rats were more risk seeking following gains (H-L) than following losses (H-Z; Figs. 2, 5, 8, and 11). Therefore, neither prospect theory nor optimal foraging theory seems able to account for the effects of previous outcomes on subsequent choice behavior in the present tasks.

Other theoretical frameworks predict greater risk seeking following gains than following losses, such as the reinforcement learning model [20] and the quasi-hedonic editing hypothesis [77–79]. The reinforcement learning model assumes that an individual maintains an expectation of choice value; any deviation from this expectation causes a prediction error that is used to update reward expectancy [67,68]. As higher valued outcomes are more likely to be chosen over lower valued ones, recent outcomes that increase or decrease the subjective value of a choice would thereby increase or decrease, respectively, the propensity to make that choice again. Thus, win-stay/lose-shift behavior is a direct prediction of reinforcement learning, consistent with the present results (Figs. 2, 5, 8, and 11). While such a prediction may depend on the assumptions of the reinforcement learning algorithm [80], the present results are considered in terms of simple model-free reinforcement learning (i.e., updating choice expectations directly based on experience with such choices), which does contribute to win-stay / lose-shift behavior in humans [80]. Accordingly, this reinforcement learning model also proposes that high-uncertainty gains and losses are gauged relative to a high-uncertainty reference point, but this is not consistent with the present findings.

In the quasi-hedonic editing hypothesis, the differences in choice behavior following prior losses and prior gains are due to the differential integration of prior outcomes with prospective outcomes. Following gains, individuals are predicted to be risk seeking because subsequent losses are perceived as decreases in current gains; alternatively, individuals are risk averse following losses due to aversion to additional losses [77]. Accordingly, win-stay/lose-shift behavior is predicted by the quasi-hedonic editing hypothesis. However, a corollary of this framework is that individuals will exhibit risk seeking behavior following losses if subsequent gambles permit the ability to “break even” [81,82]. As the rats in all groups of the present experiments seemingly had the opportunity to break even (given prior losses) by making a subsequent high-uncertainty choice (i.e., receiving the 11-pellet H-L outcome), a break-even effect would have been evident if the rats made more high-uncertainty choices following losses (e.g., H-Z outcomes) than following gains (e.g., H-L outcomes), which was not shown here (Figs. 2, 5, 8, and 11). Therefore, the proposed effects of previous outcomes on subsequent choice behavior by both the reinforcement learning and quasi-hedonic editing hypothesis frameworks can only partially account for the molecular results in the present experiments.

A more recent theoretical framework that is potentially relevant to the present studies is tri-reference point theory [19]. Rather than assuming that an individual uses one reference point to distinguish gains from losses, the tri-reference point theory assumes that individuals use three reference points that partition outcomes into failures, losses, gains, and successes. The reference points that separate failures from losses, losses from gains, and gains from successes are referred to as the minimum requirement, the status quo, and the goal reference points, respectively. Wang and Johnson [19] proposed that outcomes on opposite sides of a given reference point should have greater effects on behavior relative to outcomes between two reference points. The expected value of the low-uncertainty choice in the present experiment meets this criterion in regard to high-uncertainty gains and losses. Interestingly, the significant differences in choice behavior following H-Z and H-S outcomes in Groups 1–11 and 2–11 in Experiment 1 do not adhere to this criterion; the results may then suggest the presence of a second reference point comparable to a zero-based reference point, so that H-Z outcomes are regarded as greater losses than H-S outcomes. Accordingly, future research is warranted to investigate such a phenomenon. Ultimately, even though this framework does not seem to make any explicit predictions concerning the effects of failures, losses, gains, and successes on subsequent choice behavior, the tri-reference point theory may serve as a comprehensive approach to understanding reference points for different outcomes.

### An Integration of Theoretical Frameworks

The theories of choice behavior described above posit distinct mechanisms to account for probabilistic decision making. While each theory may not be able to fully account for the present data, there are individual elements that are applicable. For example, prospect theory suggests that the subjective value of a gain is a positively increasing, negatively accelerating function of the objective magnitude of the gain [16]. Accordingly, the subjective value of greater outcome magnitudes is increasingly diminished so that the difference between 2 and 4 pellets would be subjectively greater than the difference between 8 and 10 pellets. While this negative acceleration has been used to explain overall risk aversion in the domain of gains [16], this component of prospect theory may be able to explain the effects of the previous outcome on subsequent choice behavior. This is also consistent with both Weber’s law, which predicts that the same absolute difference in outcome magnitude would elicit smaller effects on choice behavior with increases in absolute outcome magnitude (also see [66] for an application of Weber’s law to risky choice in pigeons), as well as certain economic principles, such as diminishing marginal utility and the law of diminishing returns [16,83,84].

**Fig 12:**
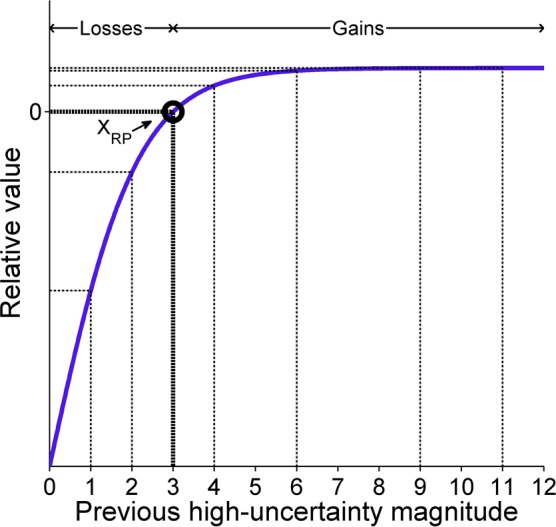
A proposed mechanism to account for the asymmetric effects of previous high-uncertainty choice outcomes on subsequent choice behavior. The abscissa is the magnitude of the previous high-uncertainty outcome. The ordinate is the subjective value of the previous high-uncertainty outcome relative to the value of the low-uncertainty outcome choice (e.g., 3 food pellets). The low-uncertainty outcome reference (*X*_*RP*_) differentiates gains and losses for the high-uncertainty outcomes.

Fig. 12 shows a diminishing marginal utility (in this case a logistic) function for the relative value of the high-uncertainty magnitudes ranging from 0 to 11 pellets, with *X*_*RP*_ demarcating the low-uncertainty reference point of 3 pellets in this example. Note that high-uncertainty outcomes greater than *X*_*RP*_ are gains and outcomes less than *X*_*RP*_ are losses, and the relative value function reflects the experienced degree of relative gain or loss. The difference between the subjective values of previous high-uncertainty losses (i.e., x < *X*_*RP*_) is greater than the differences between the subjective values of previous high-uncertainty gains, and as a result differential losses are predicted to elicit greater differences in subsequent choice behavior than differential gains (see Figs. 2 and 5). This mechanism accounts for the three novel findings in the present experiment: (1) a low-uncertainty reference point to distinguish probabilistic gains and losses (*X*_*RP*_), (2) an augmented sensitivity to losses over gains due to the steeper function associated with smaller high-uncertainty magnitudes (losses), and (3) an effect of non-zero losses distinguishable from effects of both zero-magnitude losses and non-zero gains. This model also predicts the strong effect of the low-uncertainty outcome magnitude on choice behavior that was observed in Experiment 3B (Fig. 10) as this would be equivalent to changing the value of *X*_*RP*_.

In conjunction with the present results, previous evidence supports the descriptive model diagrammed in Fig. 12. For example, this mechanism is consistent with the strong effects of reward omission found within choice and non-choice paradigms [33,61,62], as the greatest differences in subjective value relative to a low-uncertainty reference point would be produced by H-Z outcomes. Therefore, high-uncertainty gains and losses relative to the expected value of the low-uncertainty choice would ultimately result in an increased and decreased tendency, respectively, to make a subsequent high-uncertainty choice. Importantly, if win-stay/lose-shift behavior was collapsed across trials, then a higher frequency of gains should produce more high-uncertainty choices and a higher frequency of losses should produce more low-uncertainty choices. Consequently, local choice behavior could ultimately predict the global pattern of an increase in high-uncertainty choice as a function of the probability of high-uncertainty food delivery (e.g., Fig. 1) [85].

As described above, diminishing marginal utility is a critical component of the model in the gain and loss domain, as increased separation of the outcomes from the reference point should attenuate the impact of the individual outcomes. While the case could be made that the present results could be purely explained in terms of marginal utility, there does seem to be reason to believe that a reference point account is mutually exclusive from a marginal utility account. Specifically, the criterion from which utility diminishes may be regarded as an individual’s reference point. Indeed, in non-human primates, Stauffer et al. [86] demonstrated diminishing dopaminergic responses to equivalent prediction errors farther from the overall expected value of a fixation stimulus. As these corresponding data were not collected in a choice procedure, the primary reference point in that experiment should have been the expected value of the fixation stimulus. The present results complement these data by demonstrating that the primary reference point from which utility and value of high-uncertainty gains and losses diminishes is the expected value of the *low-uncertainty* choice. Specifically, as the abscissa in Figs. 3 and 6 could be relabeled “H-S prediction error”, a high-uncertainty reference point explanation, in conjunction with Stauffer et al. [86], would have predicted that an increase along the abscissa produces more similar treatment of the H-L and H-S outcomes (i.e., the H-L and H-S outcomes are farther from the high-uncertainty reference point). However, the constancy of the function suggests that despite changes in the expected value of the high-uncertainty choice, the reference point was in fact constant across conditions. Therefore, in accordance with the explanations above, our data support a low-uncertainty reference point.

## Conclusions

Probabilistic gains and losses drive the decisions among multiple choice outcomes that differ in the magnitudes and probabilities of their outcomes. Behavioral, neurobiological, and neuro-economic accounts of such effects have shaped our understanding of probabilistic decision making [16,67,87–91]. In conjunction with these contributions to our understanding of probabilistic decision making, the present experiments have provided crucial insight into the mechanisms of choice behavior that determine judgments of high-uncertainty gains and losses in rats. To our knowledge, the present set of studies may compose one of the most comprehensive behavioral accounts of trial-by-trial probabilistic choice behavior in terms of reference point use in rats, an important preclinical model for human choice behavior [15]. Accordingly, while differential sensitivities to gains and losses have been described in humans [16,18,63], there has been relatively minimal discussion of reference-point use in animals, especially regarding the general notion that high-uncertainty gains and losses are gauged relative to the expected value of the low-uncertainty choice (but see [46,92,93] for a related mechanism). The present results also have implications for the mechanisms underlying factors that influence gambling behavior, such as those involved in processing losses-disguised-as-wins [65]. Given previous reports identifying individual differences in choice behavior [52,94,95], future research should address individual differences in reference point use and how that may predict risk-taking behavior. In consideration of the present support for a low-uncertainty reference point, the possibility must be entertained that the current models and frameworks of probabilistic choice require modification. Overall, the present experiments represent a considerable advancement in forming a deeper understanding of the mechanisms of probabilistic choice behavior in animal models. Only when such a comprehensive understanding is achieved can we then begin to fully elucidate the underlying psychological and neurobiological processes that drive individual differences in the risky decision making behaviors that can plague society.
